# Animal-Mediated Ecosystem Process Rates in Forests and Grasslands are Affected by Climatic Conditions and Land-Use Intensity

**DOI:** 10.1007/s10021-020-00530-7

**Published:** 2020-08-06

**Authors:** Didem Ambarlı, Nadja K. Simons, Katja Wehner, Wiebke Kämper, Martin M. Gossner, Thomas Nauss, Felix Neff, Sebastian Seibold, Wolfgang Weisser, Nico Blüthgen

**Affiliations:** 1 Terrestrial Ecology Research Group, Department for Ecology and Ecosystem Management, Technical University of Munich, Hans-Carl-von-Carlowitz-Platz 2, 85354 Freising, Germany; 2 Department of Agricultural Biotechnology, Faculty of Agriculture, Düzce University, 81620 Düzce, Turkey; 3 Ecological Networks, Technische Universität Darmstadt, Schnittspahnstr. 3, 64287 Darmstadt, Germany; 4 Genecology Research Centre, University of the Sunshine Coast, Maroochydore DC, QLD 4558 Australia; 5 Forest Entomology, Swiss Federal Research Institute WSL, Zürcherstrasse 111, 8903 Birmensdorf, Switzerland; 6 Department of Environmental Systems Science, Institute of Terrestrial Ecosystems, ETH Zurich, 8092 Zurich, Switzerland; 7 Environmental Informatics Unit, Department of Geography, University of Marburg, 35032 Marburg, Germany; 8 Landscape Ecology, ETH Zürich, Universitätstrasse 16, 8092 Zurich, Switzerland; 9 Ecosystem Dynamics and Forest Management Group, Technical University of Munich, Hans-Carl-von-Carlowitz-Platz 2, 85354 Freising, Germany

**Keywords:** Dung removal, Seed removal, Predation, Herbivory, Grazing, Mowing, Wood harvest, Deadwood, Conifer plantations

## Abstract

Decomposition, vegetation regeneration, and biological control are essential ecosystem functions, and animals are involved in the underlying processes, such as dung removal, seed removal, herbivory, and predation. Despite evidence for declines of animal diversity and abundance due to climate change and land-use intensification, we poorly understand how animal-mediated processes respond to these global change drivers. We experimentally measured rates of four ecosystem processes in 134 grassland and 149 forest plots in Germany and tested their response to climatic conditions and land-use intensity, that is, grazing, mowing, and fertilization in grasslands and the proportion of harvested wood, non-natural trees, and deadwood origin in forests. For both climate and land use, we distinguished between short-term effects during the survey period and medium-term effects during the preceding years. Forests had significantly higher process rates than grasslands. In grasslands, the climatic effects on the process rates were similar or stronger than land-use effects, except for predation; land-use intensity negatively affected several process rates. In forests, the land-use effects were more pronounced than the climatic effects on all processes except for predation. The proportion of non-natural trees had the greatest impact on the process rates in forests. The proportion of harvested wood had negative effects, whereas the proportion of anthropogenic deadwood had positive effects on some processes. The effects of climatic conditions and land-use intensity on process rates mirror climatic and habitat effects on animal abundance, activity, and resource quality. Our study demonstrates that land-use changes and interventions affecting climatic conditions will have substantial impacts on animal-mediated ecosystem processes.

**Electronic supplementary material:**

The online version of this article (10.1007/s10021-020-00530-7) contains supplementary material, which is available to authorized users.

## Highlights

- In forests, land-use affected ecosystem processes more than climatic conditions,
- In grasslands, climatic conditions affected ecosystem processes similar to or more than land use,
- Land-use intensity had negative effects on ecosystem processes in grasslands,
- Direction and strength of climatic effects varied among habitat and process.

## Introduction

Animals are involved in various ecosystem processes underlying crucial ecological functions (Dirzo and others 2014). For example, dung removal, seed removal, and herbivory conduce to nutrient cycling and vegetation regeneration (Nichols and others 2008). Predation forms the backbone of biological control (Maas and others 2016). The rate of animal-mediated ecosystem processes—measured as the amount of resource used or the number of animals involved in a given process and time period (Meyer and others 2015)—is a direct result of the presence, behavior, and activity levels of the animals involved in these processes. Climatic conditions and land-use intensity, two major drivers of global biodiversity change (IPBES 2019), alter animal community attributes; therefore, we expect that those drivers have profound effects on animal-mediated ecosystem processes. However, the combined effects of climatic conditions and land-use intensity on animal-mediated ecosystem processes have, to our knowledge, not previously been tested. We aimed to address this knowledge gap, which enhances our understanding of the response of ecosystem functions to global change drivers.

Land-use intensity and climatic conditions can alter the rates of animal-mediated ecosystem processes by changing habitat characteristics and resource availability for animals, as well as reproduction and mortality rates (Chisté and others 2016; Mainwaring and Hartley 2016; Birkhofer and others 2017). Land-use intensification is likely to affect animal-mediated process rates via reduction in local biodiversity and biotic homogenization (Naeem and others 2012). For example, intensively fertilized and mown grasslands were found to have lower predation rates (Meyer and others 2019) due to lower plant and animal richness, respectively (Socher and others 2013; Allan and others 2014). Dung removal rates decreased with greater harvest intensity in forests but increased with higher grazing intensity in grasslands, which was mediated by the variation in dung beetle abundances (Frank and others 2017). It is, however, unknown if land-use effects are consistent among complementary processes, that is, processes that involve different animal groups.

Climatic conditions in the long term have significant positive or negative effects on process rates depending on the animal groups involved and the process under question. For example, an increase in mean annual temperature was found to be positively correlated with seed removal by invertebrates but not by vertebrates, and to herbivory in the northern but not the southern hemisphere (Peco and others 2014; Zhang and others 2016). Animal-mediated process rates are not solely driven by long-term climatic conditions, but may also vary according to short-term (that is, on day of sampling) and medium-term (that is, current and previous seasons) climatic conditions. On short-term timescales, weather conditions, such as ambient temperature, light intensity, and humidity, affect the metabolic rate of ectotherms and activity level of animals in general (Kevan and Baker 1983; Saska and others 2013); weather thereby influences how each individual performs a specific process per unit time (*per capita* process rate). Furthermore, weather conditions influence resource characteristics, such as dung moisture, and thus, resource attractiveness to animals (Edwards 1991). It is essential to understand the responses of animal-mediated ecosystem processes to conditions at different timescales, especially for determining ecosystem multifunctionality that involves assessing several processes together but at different periods (for example, Felipe-Lucia and others 2018).

Differential short- versus medium-term effects on animal-mediated processes may also be found for land use. For example, short-term effects are expected as a result of the reduction in animal densities directly after mowing in grasslands (Humbert and others 2009). Longer-term effects of land-use intensity on processes may arise from changes in habitat characteristics, which lead to changes in plant and animal communities and densities. In this paper, we examined the effect of climatic conditions and land-use intensity on animal-mediated processes along land-use intensity gradients in forests and grasslands, two major terrestrial habitats of Central Europe. Semi-natural grasslands and forests differ in plant and animal community composition, abundance, and habitat structure (Evans and others 2005). Therefore, rates of ecosystem processes and their responses to global change drivers in those habitats can differ substantially. Grassland management in Central Europe occurs at small scales and varies in intensity (Squires and others 2018). Grazing, mowing, and fertilization are the major components of grassland management (Pykälä 2001; Blüthgen and others 2012) with significant effects on plant and animal communities (Verhulst and others 2004; Dengler and others 2014; Simons and others 2015). Forests in Central Europe have been largely modified to optimize timber production (McGrath and others 2015). Three major components of forest management that shape habitat conditions are measured as the proportion of harvested tree volume, the proportion of tree species that are not part of the natural forest composition, and the proportion of anthropogenic deadwood in the total amount of deadwood. These variables have been shown to affect animal communities by modifying tree biomass, vegetation structure and composition, microhabitat conditions, and resources for animals (Müller and others 2007; Frank and others 2017; Leidinger and others 2019). Although the effects of some of these land-use components have been shown for single processes for time periods of several years (Gossner and others 2014; Frank and others 2017; Meyer and others 2019), it is not known whether those effects are consistent across short- and medium-term timescales.

We designed our study to address the following research questions:

1. Are animal-mediated ecosystem processes more strongly driven by climatic conditions or land-use intensity?
2. How important are climatic conditions and land-use intensity on a short-term timescale (during surveys in the field) in comparison to the medium-term timescale (in the preceding years)?
3. To what extent do climatic conditions and land-use intensity affect processes in forests and grasslands differently?

## Materials and Methods

### Study Regions

The study was conducted within the framework of the Biodiversity Exploratories project, where 150 semi-natural grassland plots (50 m × 50 m) and 150 forest plots (100 m × 100 m) were installed to study the effects of land use on biodiversity and ecosystem functioning (Fischer and others 2010). Plots are located in three regions of Germany, spanning a climatic range from oceanic to continental: the UNESCO Biosphere Reserve Schorfheide-Chorin (SCH) in northeastern Germany, the National Park Hainich and surroundings in central Germany (HAI) and the UNESCO Biosphere Reserve Schwäbische Alb (ALB) in southwestern Germany. Elevation ranges were 3–140 m above sea level (a.s.l.) for SCH, 285–550 m a.s.l. for HAI, and 460–860 m a.s.l. for ALB (Fischer and others 2010). The plots have homogenous and continuous vegetation cover and represent the typical soil conditions, major grassland or forest vegetation types, land-use types, and intensities of each region (Fischer and others 2010). Land use in grasslands ranged from extensively managed (that is, typically non-fertilized meadows mown once annually and pastures grazed only briefly by sheep or cows) to intensively managed meadows (that is, fertilized and mown up to five times annually and pastures with more than 30 cattle grazing continuously) (Vogt and others 2019). Forests in the regions included even-aged managed conifer plantations (of *Picea abies* and *Pinus sylvestris*, which are considered non-natural to the study sites), unmanaged or managed beech-dominated stands (*Fagus sylvatica*) with uneven or even age structure, even-aged managed oak stands (*Quercus* spp.), and forest stands co-dominated by beech and pine trees (Kahl and Bauhus 2014).

### Quantifying Process Rates

**Figure 1:**
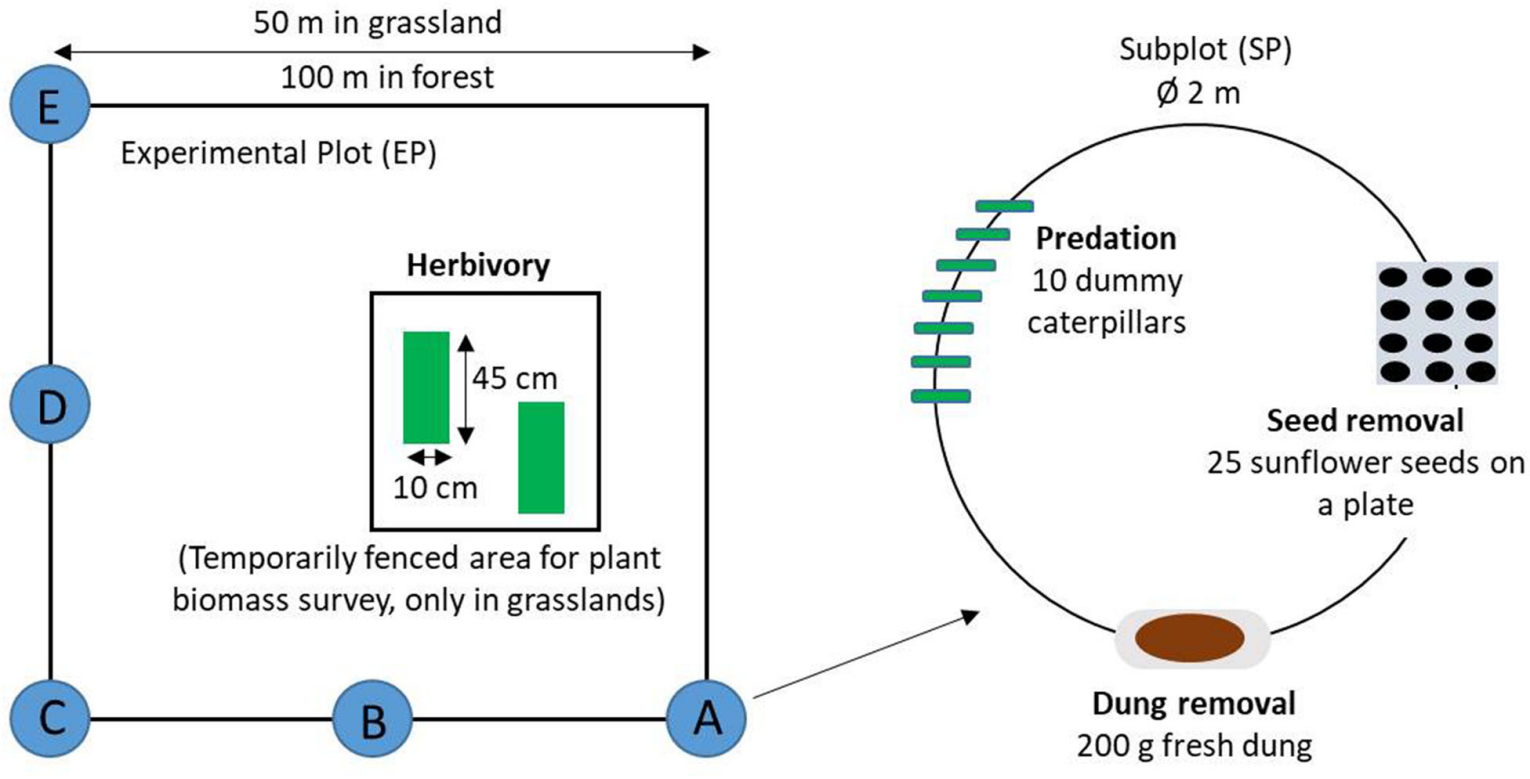
Experimental setup for measuring ecological processes in each experimental plot with five subplots indicated with letters. In forests, herbivory was assessed on the dominant plant species, which were sampled along the border of the plot (not shown, see supplementary material S2 for the details).

In 2017, we conducted surveys of dung removal, seed removal, predation, and foliar herbivory by arthropods (hereafter referred to as “herbivory”), adapted from rapid ecosystem function assessment methods (Gossner and others 2014; Meyer and others 2015). Dung removal, seed removal, and predation were measured in 50 forest and 50 grassland plots at ALB and HAI and in 49 forests and 34 grassland plots at SCH. Herbivory was assessed on 50 plots per region and habitat type. To measure dung removal, seed removal, and predation, we set up five regularly placed circular subplots (2 m in diameter) along the south and west edges of each plot (Figure 1). Surveys were conducted in each plot for 48 h in all three regions during June 2017. Herbivory in grasslands was measured on biomass samples taken from two other subplots within the same plots as in May 2017; in forests, biomass was taken from samples of the 10 most dominant plant species of each plot in June–August 2017 (for details, see below).

*Dung removal:* We measured the dung removal rate as the proportion of cattle dung removed within 48 h. The method was shown to reflect the activities of tunneling (paracoprid) dung beetles (Frank and others 2017). Cattle dung was obtained from Oberfeld, an organic farm in Darmstadt, Germany. At each subplot, approximately 200 g (exact weight was noted) of frozen dung was placed on cellulose paper tissue and left on the ground (Figure 1). After 48 h, the remaining dung was collected, dried at 60°C for 5 days, and reweighed. The dry weight of the dung samples ranged between 14.48 g and 48.74 g (mean 28.43 g, *N *= 1409). To calculate the amount of removal, we first calculated the proportion of dry mass in fresh dung (*p*_dry_ = 0.18) of randomly selected samples. Fresh weight (*fw*_before_) of each sample at the beginning of the survey was then converted to dry weight (*dw*_before_) as *dw*_before_=* fw*_before_ * *p*_dry_. The proportional removal rate (*r*_dung_) was calculated as *r*_dung_ =* (dw*_before_ −* dw*_after_)/*dw*_before_. Holes in the underlying cellulose tissue were used to verify the activity of tunneling dung beetles (Geotrupidae and *Onthophagus*) (Frank and others 2017). In 11 of 1409 subplots (0.8%), we did not find any holes; these subplots were therefore not confirmed for tunneling dung beetle activity. Five of the subplots with no confirmed tunneling dung beetle activity were assumed to have *r*_dung_=0. The other six plots showed an average *r*_dung_ = 0.144 but were excluded from the analysis because the loss could not be attributed to dung beetles.

*Seed removal:* We measured the seed removal rate by placing 25 non-viable (microwaved) intact sunflower seeds on plastic trays with 25 dents to reduce loss by wind (Figure 1) (Meyer and others 2015). The seeds remaining after 48 h were counted, and the proportion of removed seeds was used as a measure of absolute seed removal rate. Seeds were occasionally washed from the trays by rain; therefore, we decided to omit data from sites with total precipitation greater than 30 mm during the 48 h survey when most of the seeds disappeared (*N *= 18 sites, Figure S1). The precipitation threshold was assigned based on inspection of graphs showing removal rate versus total precipitation over 48 h.

*Predation*: To measure predation by arthropods and vertebrates, we exposed caterpillar dummies made from green plasticine (Noris Plasticine, Staedtler, Germany) on the ground and assessed attack rates from marks on the dummies (Figure 1) (Howe and others 2009). On each subplot, ten dummy caterpillars were pinned to the ground (spaced 10 cm apart). After 48 h, caterpillars were collected and the number of missing caterpillars was recorded. In the laboratory, marks on the caterpillars were identified using a stereo-microscope and attributed to different animal groups using templates (Low and others 2014; Meyer and others 2017): Arthropod attacks were identified by mandibular, cheliceral, and stylet marks; rodent attacks had tooth marks; bird attacks had beak marks; and gastropod attacks had radula marks. We excluded marks of gastropods from the analysis (*N *= 2759 dummies out of 13,735) because gastropods are opportunistic scavengers, not predators (Meyer and others 2019). Furthermore, we did not include missing caterpillars because predation attempt cannot be confirmed with attack marks. Therefore, predation rates were calculated by dividing the number of caterpillars with at least one attack mark by the total number of caterpillars that were recovered in the field. We calculated predation rates with this approach for overall predation, considering marks irrespective of the animal group, as well as for predator-group-specific rates.

*Foliar herbivory by arthropods:* We measured the herbivory rate by using different sampling protocols in each habitat type. In grasslands, herbivory assessments were based on biomass samples from two subplots (Figure 1). The subplots were located within a temporary exclosure to prevent vertebrate grazing and mowing. The samples were taken in May 2017. A metal frame of 10 cm × 45 cm × 2 cm was placed randomly at two sampling locations in the exclosure (Figure 1). The vegetation was cut along the edge of the frame at 2 cm height. Herbivory rates were assessed on 100 randomly selected leaves. Grasses and herbs were represented in proportion to their contribution to the total biomass. In forests, we estimated the herbivory rate on the most abundant plant species. We selected these species based on plant cover data from previous years (Table S1; Boch and others 2013; Daniel Prati pers. comm). The leaf material was collected from the outer border of the plots in summer (mid-June and early July in ALB, end of July to mid-August in HAI, and July to August in SCH). For samples from both habitat types, the damaged area on each leaf was estimated visually by comparing the damaged leaf area to a series of circular and square templates ranging in size from 1 to 500 mm^2^ (Gossner and others 2014). We included different damage types such as chewing, sap sucking, leaf mining, and rasping (Loranger and others 2014). The remaining area was measured by a leaf area meter (LI-COR area meter, LI-3100C, Lincoln NE, USA). Herbivory rates were calculated as a proportion by dividing the total damaged area by the total leaf area in the sample. Community-level herbivory rates on grasses, herbs, or total herbivory were used as response variables for grasslands. For forests, the herbivory rates across all assessed plant species were weighted by plant cover per plot (see details of the herbivory assessment in Supplementary Material S2).

### Climate Variables

For climatic conditions, the short term refers to the conditions during survey periods. For dung removal, seed removal, and predation rate measurements, the short-term period was 48 h. To quantify short-term climatic conditions, we summed the total daily precipitation (mm) and calculated mean temperature of the short-term period. We used the mean of either the daily mean or daily maximum temperature (°C), depending on the explanatory power of the variables on each process (see data analysis section). For the short-term effects on herbivory, we calculated the climate variables during the period when arthropod herbivory on the leaves in the biomass samples took place: from the start of the vegetative season (March 29, 2017) until the date of sampling in each plot. For herbivory, we used the sums of daily precipitation and temperature because our measurements revealed the cumulative result of herbivory that took place over weeks (see Supplementary Material S3 for additional details of explanatory variables and time scales).

To quantify medium-term climatic conditions, we focused on the conditions of the preceding 2 years. We regarded spring and summer as the warm period for each year and autumn and winter as the cold period (September 20, 2016–March 28, 2017). For the warm period, we used the sum of daily precipitation, the mean of maximum daily temperature, and the number of cool days (that is, days with a maximum temperature of 10°C or lower) in 2016 and 2017, as separate variables. Daily precipitation and temperature variables reflect the suitability of climatic conditions for vegetation growth, animal survival, and reproduction. We assessed the number of cool days in a warm period, because this may affect the survival and reproduction of ectotherms (Retana and Cerdá 2000) as well as vertebrate juveniles (Robinson and others 2007). For the cold period of 2016–2017, we used the lowest value of the daily minimum temperatures of September and across the cold period, separately. We set the start and end of each period based on the start or end of successive frost days (a day with an hourly temperature below 0°C), which marks the start and end of the growing season. Climate data were measured by climate stations installed at each plot (see Supplementary Material S3 for details).

### Land-Use Variables

We selected three variables to quantify land-use intensity in grasslands: the intensity of livestock grazing (livestock unit days/ha; hereafter grazing), the frequency of mowing (number of cuts), and the amount of fertilization (kg nitrogen/ha) (Fischer and others 2010; Blüthgen and others 2012). We also tested for the effect of days since the last mowing, but that variable did not significantly improve the explained variation in our generalized linear mixed-effects models (data not shown).

We used three major components of forest management intensity for each forest site as explanatory variables: (1) the proportion of harvested tree volume (measured as the volume of harvested timber divided by the sum of the volume of living trees, harvested timber, and deadwood per forest, hereafter called wood harvest), (2) the proportion of tree species that are not part of the natural forest composition (measured as the volume of standing timber, harvested timber, and deadwood of non-natural trees, including spruce and pine, divided by the sum of the volume of all tree species, hereafter called non-natural trees), and (3) the proportion of anthropogenic dead wood showing signs of saw cuts (measured as the volume of deadwood with saw cuts divided by the total volume of deadwood, hereafter called anthropogenic deadwood) (Kahl and Bauhus 2014).

Unlike climatic conditions, land-use activities in plots only occur sporadically. Therefore, we followed the same short- versus medium-term logic to quantify the land-use intensity but used different time periods. To quantify the short-term effects of grassland management, we summed up the management activities that occurred from the beginning of the year until the date of surveys at each site (May or June 2017). For the medium-term effects of each land-use component, we obtained the intensity standardized relative to its mean within that year and then calculated the average of two preceding years (2015 and 2016). The surveyed forests are not managed every year and the latest measurements of management intensity was in 2012; thus, we only tested medium-term land-use effects in forests where the intensity takes the potential cumulative merchantable volume of the last 30–40 years into account (Kahl and Bauhus 2014).

### Data Analysis

All analyses were conducted in R version 3.4.3 (R Core Team 2017). To avoid potential type II errors, we checked collinearity by using correlation tests and the “corvif” function; we did not include variables with variance inflation factors greater than four in the models (Zuur and others 2009). Among the climate variables that we selected to test on the dung removal, seed removal, and predation rates, short-term variables were weakly correlated (*r* < 0.25), but several of the medium-term climate variables had higher correlation coefficients (*r* > 0.40) (Supplementary Material S3). We, therefore, conducted a principal component analysis and extracted the scores of the first two axes (PCA1 and PCA2) via the factoextra package (Kassambara and Mundt 2017) and used these scores as explanatory variables in our tests. Together, PCA1 and PCA2 explained 80% of the variation (see Supplementary Material S3). The first axis represented higher temperatures and less rain in the warm period of 2016 (referred to as dry summer). The second axis represented lower minimum temperatures in September and during the cold period (referred to as cold winter). We applied the same approach to climate variables used in the herbivory analyses, where the second PCA axis represented the warm conditions in the cold period (referred to as warm winter, Supplementary Material S3). This resulted in correlations between the short- and medium-term climate variables (Supplementary Material S3); therefore, we excluded short-term precipitation from the overall herbivory analyses.

Similarly, there were high correlations (*r* > 0.45) between intensities of specific land management variables in short- versus medium-term, and between medium-term intensities of different variables (Supplementary Material S3). Therefore, we included only the variables of the short-term land use in the basic models, whose effects are largely unknown. Medium-term land-use intensities were added to the tests and maintained only if they contributed to significantly lower deviance (tested with ANOVA). For herbivory in grasslands, medium-term fertilization intensity was excluded from the analyses because it was strongly correlated with short-term fertilization intensity (*r* = 0.66). Therefore, we tested the effect of grazing and mowing intensities at the medium timescale, but for the short-term effects, we analyzed only fertilization and grazing intensity, since no mowing took place in the period leading up to the surveys. Data from three plots were not used because either grazing or mowing had likely happened on the sampling plot.

We used generalized linear mixed-effects models (GLMM) to test for effects of climatic conditions and land-use intensity on the rates of dung removal, seed removal, and predation at a subplot level (5 per plot). For herbivory, we used plot-level data. We accepted results as significant at an alpha level of 0.05 for all tests. We further indicated trends that were close to significance, where *p* ≤ 0.1. We included short-term and medium-term climate variables and land-use variables as predictors and tested for interactions between the climate variables. We included plot nested within region (1|Region/Plot) as a random effect to account for the nested design.

The basic model structure for grasslands was:

*Rate* ~ short-term precipitation + short-term temperature + interaction of short-term climate variables + medium-term climate PCA axis 1 + medium-term climate PCA axis 2 + interaction of medium-term climate variables + short-term grazing + short-term mowing + short-term fertilization + (1|Region/Plot).

The basic model structure for forests was:

*Rate* ~ short-term precipitation + short-term temperature + interaction of short-term climate variables + medium-term climate PCA axis 1 + medium-term climate PCA axis 2 + interaction of medium-term climate variables + wood harvest + non-natural trees + anthropogenic deadwood + (1|Region/Plot).

For each process rate, we first tested the effect of the mean of the daily maximum or daily mean temperature as a short-term variable in competing models. We then selected the climate variable in the model with the lowest deviance and used it in the final model. As a result, we used the mean of the daily maximum temperature as an explanatory variable for the rate of dung removal, seed removal, and herbivory, but daily mean temperature for predation rate. We used a Gaussian distribution with a log link function for dung removal and herbivory rates. To avoid log(0), we added 1 to the all dung removal rates. For seed removal and predation rates, we used a binomial error structure and analyzed the response as the number of removed seeds vs non-removed seeds and attacked vs non-attacked caterpillars with the cbind function. To allow comparison of effect sizes, we standardized the quantitative explanatory variables to mean = 0 and standard deviation = 1. We used the lme4 package for fitting the models (Bates and others 2015). In all models, we used the bobyqa optimizer, with one million iterations, to avoid convergence problems. We did not do any model simplification because we wanted to assess the effect of each variable in relation to others, for example, climate versus land use.

**Figure 2:**
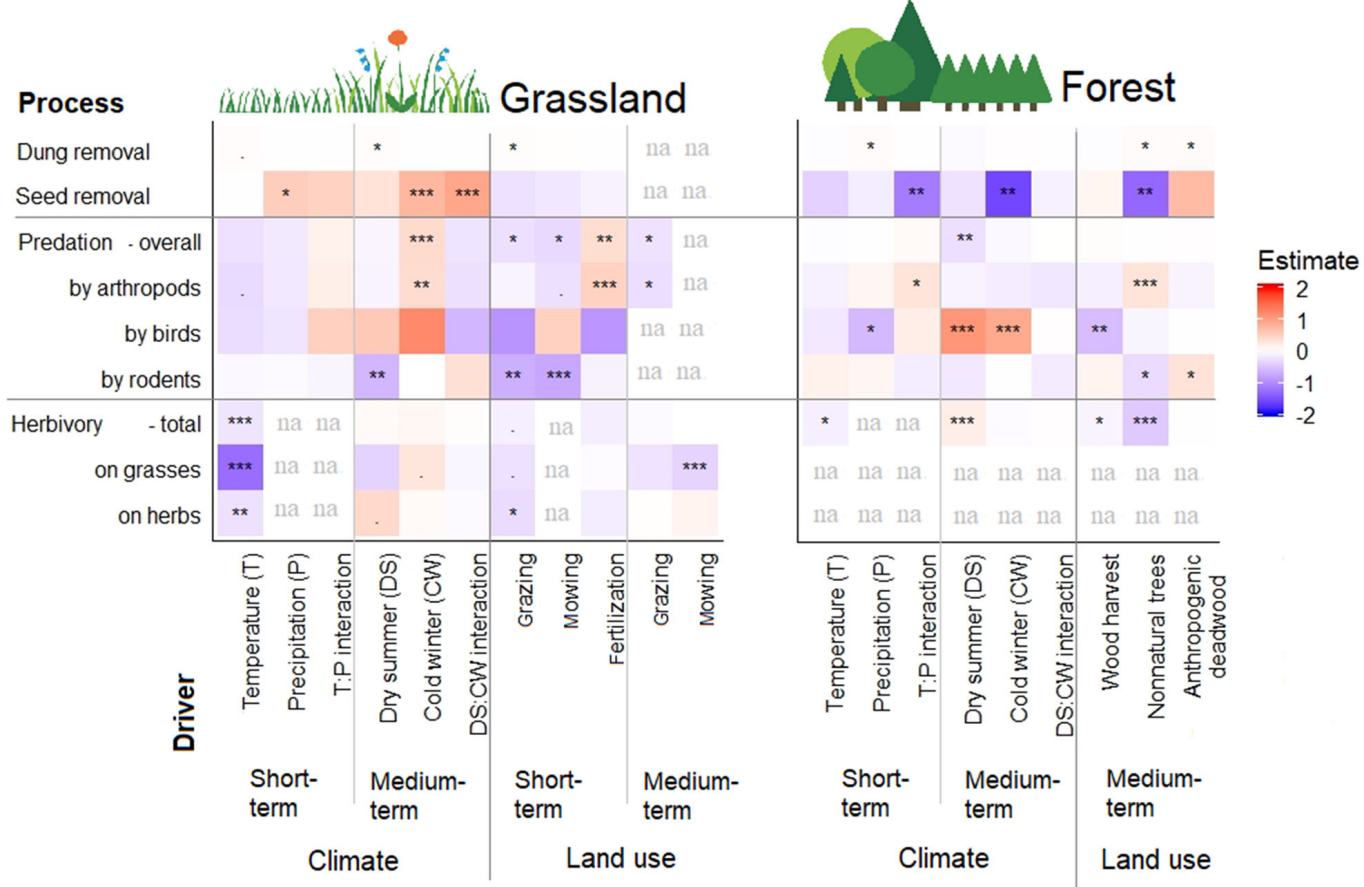
Heatmaps summarizing the effects of short- and medium-term variables of climatic conditions and land-use intensity on process rates in grasslands and forests. The color in each grid represents effect sizes (estimates from standardized variables) obtained from GLMM. Each row represents one model. Parameter significance indicated as *p ≤ 0.05, **p ≤ 0.01, ***p ≤ 0.001. We further indicated trends close to be significant with “.”*p* ≤ 0.1. Positive effects are in red (including significant but weak effects on dung removal), and negative effects are in blue. Effect sizes very close to zero (very small effects) appear in white. Heatmaps are divided by solid lines for the ease of separating processes vertically and the driver sets horizontally. Note that the short-term temperature variable implies the mean of daily maximum of the survey period for seed removal, dung removal and herbivory, but the mean of daily mean temperatures for predation (see methods). The short-term temperature variable used in herbivory analyses was the cumulative of the maximum daily temperature for the survey period. Estimates of medium-term climate PCA2 for herbivory analyses were reversed to have the same gradient of PCA2 for other processes. Processes not measured in a habitat or variables that were not tested are indicated as na.

To calculate the overall effect of a variable set on process rates (for example, climatic conditions versus land-use intensities), we used the effect sizes and the standard errors estimated by each model and calculated the weighted mean, that is, the absolute value of the effect size of each variable in a set was weighted by its standard error before calculating the mean of that set. For the comparison of climatic conditions versus land-use intensity and short- versus medium-term climatic conditions, we used the results presented in Figure 2 and Supplementary Material S4. We compared the short-term versus medium-term effects of land-use intensity in grasslands only because the forest management activities in our study took place only within medium-term timescales. Not all of the land-use variables in grasslands were included in the same models; therefore, we then ran two sets of additional models: one with climatic conditions and short-term land-use intensities and one with climatic conditions and medium-term land-use intensities. We subsequently extracted the effect sizes of the land-use variables to calculate and compare the mean effect size for short-term versus medium-term land-use effects (see Supplementary Material S5 for details).

## Results

### Process Rates in Grasslands and Forests

At the plot level, considering both grasslands and forests together, an average of 41 ± 4.2 g dung was removed from an average of 142 g dry dung, 95 ± 3.9 out of 125 seeds were removed, and 19.5 ± 1.5 caterpillars were attacked out of an average of 48.5 recovered dummy caterpillars in 48 h (see Supplementary Material S6 for recorded process rates in detail). In grasslands, arthropods caused attack marks in 85% of the attacked dummy caterpillars, followed by birds (10%) and rodents (10%) (Figure S21). In forests, arthropods caused attack marks in 55% of attacked dummy caterpillars, followed by rodents (48%) and birds (10%). The average herbivory rate was 2.8 ± 0.09% of the leaf area.

### Key Drivers of Process Rates

All tested climatic and land-use drivers had an effect on rates of at least one ecosystem process, but each process was influenced by different drivers (Figure 2, see Supplementary Material S4 for the full statistical results). Here, we describe the effects of key drivers, which are further illustrated as those with the largest effect size on each process in Figure 2. Dung removal was highest in grasslands with drier spring and summer conditions (medium-term climate PCA axis 1, effect size, and standard error = 0.031 ± 0.014) and in grasslands that were grazed intensively before the survey (0.026 ± 0.013). The highest dung removal rates were recorded in forests with higher management intensity, as indicated by higher proportions of anthropogenic deadwood (0.039 ± 0.017) and non-natural trees (0.041 ± 0.018). Seed removal was highest in grasslands with lower minimum temperatures in September and the winter (cold winter—medium-term climate PCA axis 2, 0.749 ± 0.216), but lowest in forests with similar conditions (− 1.587 ± 0.529), especially if forests had a higher proportion of non-natural trees (− 1.312 ± 0.436). Furthermore, vegetation height in grasslands had a significant effect on seed removal rates in grasslands (Table S41).

Overall predation was highest in grasslands with colder winter temperatures (0.376 ± 0.125) and in grasslands that were fertilized intensively before the survey (0.350 ± 0.121). Predation rates were low in forests with drier summer conditions (− 0.272 ± 0.088). Furthermore, vegetation structure had a significant effect on predation rates driven by the arthropods in both habitats (Table S41 and Table S42).

Arthropod-related predation was highest in grasslands with lower minimum temperatures in the previous winter (0.363 ± 0.135) that were recently fertilized (0.475 ± 0.130). Arthropod-related predation was highest in forests with a higher proportion of non-natural trees (0.285 ± 0.085), but lowest in forests on cold and rainy days (0.287 ± 0.141, Figure S18).

Our selected drivers did not explain the variation in rates of predation by birds in grasslands. Predation by birds was highest in forests with drier summers (1.077 ± 0.199).

Predation by rodents was lowest in grasslands with drier summers (− 0.624 ± 0.205) that were grazed (− 0.685 ± 0.242) or intensively mown prior to the survey (− 0.747 ± 0.217). It was lowest in forests with higher proportions of non-natural trees (− 0.306 ± 0.131) but positively correlated to proportions of anthropogenic deadwood (0.301 ± 0.121).

Herbivory rates were lowest in warmer sites between March and May 2017 in both habitat types (grasslands − 0.223 ± 0.066; forests − 0.139 ± 0.054). In grasslands, herbivory rates on herbs were higher at sites where grazing intensity in the vicinity was low (− 0.304 ± 0.152). Herbivory rates on grasses were lower at sites that had intensive mowing in the medium term (− 0.367 ± 0.108). Herbivory in forests decreased with the proportion of non-natural trees (− 0.460 ± 0.068).

Among the four major processes in each habitat, only two correlations were found: seed removal in forests was negatively correlated with dung removal (Pearson’s *R *= − 0.26, *p *= 0.002) but positively correlated with overall predation (*R *= 0.32, *p* < 0.001); supporting the findings above on differential effects of drivers on each process (supplementary material S8).

### Land-Use Versus Climatic Drivers

**Figure 3:**
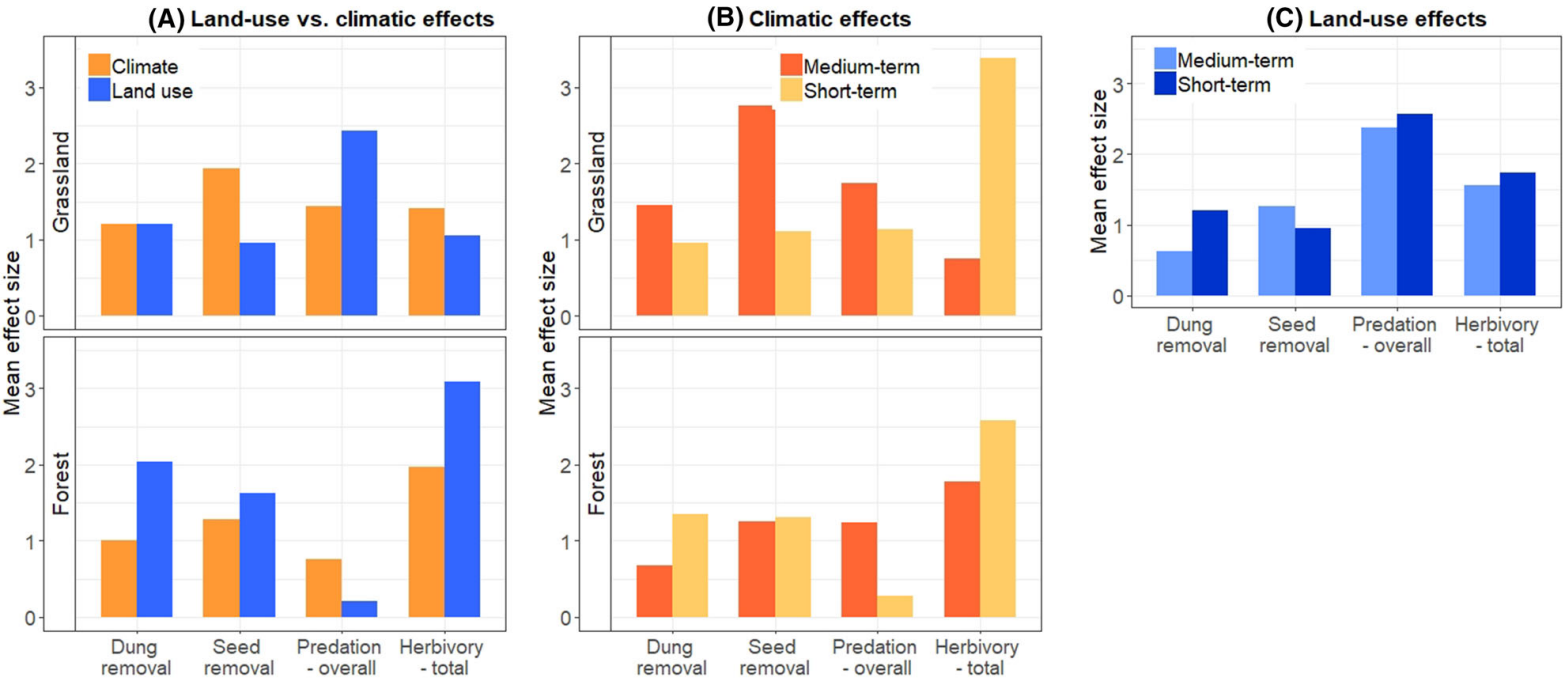
Mean effect size of climatic conditions versus land-use intensity on each process. **A** Mean effect size of all climatic versus land-use variables in two habitat types, **B** mean effect size of medium- versus short-term climatic variables in two habitats, **C** mean effect size of short-term versus medium-term land-use intensities in grasslands. The mean effect sizes were calculated per each variable group as the mean of the effect sizes (estimates from the mixed models with standardized quantitative variables) of all variables in this group weighted by the inverse of their standard error. Note that additional analyses were conducted to find our effect sizes of the short- versus medium-term land-use intensities separately (see supplementary material S5).

Based on the mean of effect sizes, the climatic effects were similar or stronger than the land-use effects in grasslands for all processes except for overall predation (Figure 3A). In forests, land-use intensity was a stronger driver than climatic conditions for all processes except for overall predation.

### Short-Term Versus Medium-Term Effects of Climatic Conditions

The short-term climatic conditions in grasslands had weaker effects on process rates (except for herbivory) than the medium-term conditions (Figure 3B). In forests, the short-term climatic conditions had similar or stronger effects than the medium-term on all process rates except for overall predation. In both habitats, short-term climatic conditions varied more strongly across plots than medium-term climatic conditions. For example, in forests, the variation coefficient of the short-term temperature was 13% compared to a maximum of 8% for the medium-term (Tables S8 and S9).

At the short-term timescale, the temperature did not have a significant effect on the processes other than herbivory, which decreased in both habitats with the sum of daily maximums (Figure 2). Short-term precipitation in grasslands was positively correlated with seed removal. In forests, short-term precipitation increased dung removal and decreased predation by birds. When precipitation was low, short-term temperature in forests increased the rates of seed removal and decreased the predation by arthropods. Short-term temperature decreased seed removal and increased predation by arthropods when precipitation was high (Supplementary Material S4).

At the medium-term timescale in grasslands, drier summer conditions (climate PCA axis 1) increased dung removal rates but decreased predation by rodents (Figure 2). In this habitat, seed removal and predation by arthropods increased with cold winter (PCA axis 2). In forests, drier summer conditions decreased overall predation but increased predation by birds and total herbivory. Cold winter decreased seed removal and increased predation by birds in forests. Herbivory rates in grasslands and forests were not affected by cold winter conditions (Figure 2). In summary, the climatic conditions had significant effects on the rates of each process, but the effects varied in direction and strength.

### Short-Term Versus Medium-Term Effects of Land-Use Intensity

The mean effect sizes of short-term land-use variables were similar or higher than that of the medium-term land-use variables in grasslands for all processes except for seed removal (Figure 3C). Furthermore, specific predation rates by birds and rodents as well as herbivory on grasses and herbs were influenced more by short-term land-use intensity than medium-term (Supplementary Material S5). In grasslands, short-term effects of land-use intensity were mostly negative, except for grazing effects on dung removal and fertilization effects on arthropod-related predation rates (Figure 2). Medium-term grazing and mowing, when included in the models, added to negative effects land use at the short-term timescale. Grazing and mowing intensity in the short term varied more compared to the medium-term (Figure S11).

We tested the effect of forest management activities only at the medium-term timescale. The proportion of harvested wood negatively affected the rates of total herbivory and predation by birds (Figure 2). The proportion of anthropogenic deadwood had positive effects on dung removal rates and predation by rodents. Dung removal rates and predation by arthropods were also positively correlated to the proportion of non-natural trees. In contrast, seed removal, predation by rodents, and total herbivory were negatively correlated to the proportion of non-natural trees (Figure 2). Among forest management components, the proportion of non-natural trees varied more across plots (standard deviation SD = 0.40) than harvest intensity (SD = 0.25) and anthropogenic deadwood (SD = 0.26). In summary, the effect of forest management varied in direction depending on the management component and the process.

### Differences in Responses Between Grasslands and Forests

The effect sizes of single drivers (Figure 2) or different driver sets (Figure 3) were not consistently stronger or weaker across habitats. In grasslands, climatic conditions had higher effect sizes than land-use effects. In this habitat, medium-term climatic conditions had higher effect sizes than those of the short-term, and short-term land-use intensity had higher effect sizes than those of the medium-term. In forests, land-use effects were stronger, and the short-term climatic conditions had similar or higher mean effect sizes compared to those of the medium-term.

The only climatic variable with consistent effects in direction was the negative effect of short-term temperature on herbivory (Figure 2). Seed removal was the only process that responded strongly to climatic conditions in two habitats, but it responded in opposite ways: positive in grasslands but negative in forests. For the other processes, the climatic drivers had a significant effect in one habitat but not in the other, for example, a significant positive effect of cold winter on arthropod-related predation rates occurred in grasslands but not in forests.

The rates of each process were higher in forests compared to grasslands (ANOVA, each *p* ≤ 0.02, supplementary material S6), except for predation by arthropods (*p* = 0.16). The two habitats also differed in the amount of variation of process rates at the subplot level. In grasslands, we found higher within-plot variation in process rates (ranging between 37 and 177%, supplementary material S9). Within-plot variations of some process rates were significantly correlated to land-use intensity (Table S46). Dung removal rates were more stable (lower within-plot coefficient of variation, CV_within_) in sites with high short-term land-use intensity (Pearson’s *R *= − 0.176, *p *= 0.04). Seed removal rates were more variable both with high short- and medium-term land-use intensity (Pearson’s *R *= 0.340, *p *< 0.001, Pearson’s *R *= 0.236, *p *= 0.008; respectively). Predation by arthropods was more stable with high medium-term land-use intensity (Pearson’s *R *= − 0.211, *p *= 0.019). We found less within-plot variation of process rates (19–130%) and no correlations of CV_within_ to management intensity in forests (Table S45 and Table S46). The habitats also differed slightly in relation to the climatic variation. Short-term climatic conditions were slightly less variable in forests compared to grasslands (CV of the precipitation sum were 154% and 162% and CV of the maximum temperatures were 13% and 15% for forests and grasslands, respectively). Medium-term climatic conditions were generally more variable in forests than grasslands, but specific variables differed in their variation across habitats (Table S8 and Table S9).

## Discussion

Our results show that climatic conditions and land-use intensity in the short-term (during survey periods) and the medium-term (preceding years) influence the rates of animal-mediated ecosystem processes in forests and grasslands of central Europe. Each predictor variable had a significant effect on at least one ecosystem process, but the effects varied between the processes, habitats, and timescales. The effects of climate or land-use variables at the short- or medium-term timescales were, therefore, inconsistent among complementary animal-related ecosystem processes.

### Land-Use Versus Climatic Drivers

We found that neither of the two sets of drivers had consistently stronger effects. In grasslands, the climatic drivers had stronger effects on process rates than land-use intensity, except for overall predation. In forests, management intensity affected process rates more strongly than climatic conditions, except for overall predation.

In grasslands, the stronger climatic effects compared to land-use effects are likely to be caused by higher variation in the abundance and/or activity of the involved animals in response to climatic conditions. For example, dung beetle densities fluctuate strongly in response to daily changes in temperature and rainfall (Finn and others 1998) and conditions during the growing season (Lumaret and others Lumaret et al. 1992). In comparison, dung removal rates were shown to have varied relatively less with grazing intensity, despite an increase in beetle biomass (Frank and others 2017). This echoes our finding that dung removal in grasslands is influenced more by climatic conditions than land-use.

Seed removal in grasslands responded to several climate variables but none of the land-use variables. This effect was probably mediated by vegetation height, which is a known driver of granivore activity (Hulme and Kollmann 2005) and was a significant predictor of seed removal rates in grasslands (Table S41).

Herbivory in grasslands was driven by both climatic and land-use effects, which may have acted directly on herbivore abundances as well as indirectly through the availability of plant resources. Previous studies have shown that land-use intensity and climatic conditions can separately change herbivore abundance by about 50% (Frampton and others 2000; Gossner and others 2014). Moreover, dry season causes a decrease in plant palatability, which can decrease herbivory and exacerbate the effects of climatic conditions (Zvereva and Kozlov 2006).

Contrary to the other processes in grasslands, we found that predation by arthropods responded more to land-use intensity than climatic conditions. This was due to arthropod predators, which were responsible for the majority of the attacks, being very sensitive to land-use effects. Specifically, mowing kills many arthropod predators and their herbivore prey (Humbert and others 2009). Grazing and mowing can also have indirect effects through vegetation structure—shorter vegetation supports a smaller predator density (Simons and others 2014; Chisté and others 2018; Meyer and others 2019). Based on this information, our finding as the positive effect of vegetation height on predation by arthropods in grasslands was not unexpected (Table S41).

In forests, six climate variables had small effect sizes with large standard errors on the process rates, whereas three components of forest management had strong significant effects on the processes (supplementary material S4). Responses of the processes to forest management were mostly due to tree composition. The dominance of non-natural conifers usually corresponds to changes in animal community composition and abundance. For example, forests dominated by conifers in the Schorfheide region had 3–4 times greater dung beetle biomass (Frank and others 2017). Moreover, conifer forests may be associated with lower rodent densities (Jędrzejewski and Jędrzejewska 1996), because they offer no beechnut or acorns as food sources. This may have led to lower rates of seed removal and rodent predation (Figure 2). Conversely, conifer forests showed a greater predation rate by arthropods, which can be a result of elevated richness and differences in the composition of some arthropod groups (Lange and others 2014; Penone and others 2019). Reduced herbivory in conifer stands can also be explained by lower herbivore abundance, but particularly by the fact that gymnosperms attract herbivores much less than angiosperms (Turcotte and others 2014).

### Short-Term Versus Medium-Term Effects

#### Short-Term Versus Medium-Term Effects of Climatic Conditions

Medium-term climatic conditions had stronger effects on the process rates than short-term conditions in grasslands, whereas the opposite was found in forests. Medium-term climatic conditions act on animal densities in general (Bale and Hayward 2010), whereas short-term conditions affect animal activity levels (Kevan and Baker 1983; Saska and others 2013). Therefore, we conclude that variations on animal densities should have had an overriding effect on the process rates compared to the fluctuations in animal activity levels in the short term. In forests, we did not observe as strong an effect of medium-term climatic conditions; instead, management and tree composition should have had a greater influence on animal abundances.

Interestingly, short-term temperature did not have a positive effect on the process rates. We expected this effect because higher temperatures increase the metabolic rate of ectothermic arthropods and their activity (Huey and Kingsolver 1989; Kühsel and Blüthgen 2015). Our results are likely due to the overriding effect of variation in abundances between the sites, whereas variation in the animals’ *per capita* contribution to processes due to momentary temperature could have played a relatively minor role.

Short-term precipitation had both positive and negative effects on the process rates. We found a positive effect of precipitation on dung removal even though rain has been reported to decrease the flight activity of dung beetles (Finn and others 1998). The positive effect is probably due to moist dung emitting attractive volatiles for a longer period (Frank and others 2018). We did not detect any leaching effect around the papers underlying the dung baits, and, therefore, we assume that the leaching effect of rainfall on dung samples is negligible. The positive effect of precipitation on seed removal could be due to gastropod seed predators, who are known to increase activity during cooler periods following rain showers (South 2012). Although we excluded samples when total rainfall exceeded 30 mm, we cannot rule out a wash-away effect of rainfall less than 30 mm on seed removal. Birds are known to be less active on rainy days (Robbins 1981), and we found supporting evidence for this through lower bird predation rates in forests. In summary, the mechanisms behind the effects of short-term climatic conditions on process rates may be linked to both animal behavior and resource quality.

We found significant effects of medium-term climatic conditions on the process rates in grasslands and forests. Medium-term climatic conditions may directly influence variation in animal abundances or indirectly affect animal communities by changes to vegetation (Oesterheld and others 2001). For example, we found a negative effect of dry summer on arthropod-driven overall predation rates in forests (Figure 2). A dry summer causes lower herbaceous vegetation height and cover, which decreases arthropod predator abundances and rodent activity levels and, therefore, the animal-mediated process rates (Table S42; Jędrzejewski and Jędrzejewska 1996). Conversely, the same conditions can help visual predators such as birds to detect prey more easily and contribute to higher predation rates (Andersson and others 2009), but we found no evidence for the indirect effect of ground vegetation cover on bird predation in forests (Table S42). Cold winter had both positive and negative effects on different process rates. A colder winter may decrease populations of some freeze-intolerant overwintering animal species (Verdú and others 2010), but some diapausing animals benefit from colder conditions; warmer winter conditions can disrupt diapause in insects and increase mortality risks due to lack of food and pathogen pressure (Harvell and others 2002; Johnson and others 2010). For rodents and some birds, warm winters and less snow may have negative effects due to increased pressure from predators or lack of food (Stenseth and others 2002).

In forests, we observed opposite effects on herbivory of the climatic variables at different timescales; in the short term, the sum of daily temperatures had a negative effect, whereas in the medium term, dry summer conditions had a positive effect. The two variables may have acted on different components of this process because higher temperatures in the short term have been shown to decrease leaf quality and digestibility (Dury and others 1998); however, a dry summer promotes populations of some herbivores, which can be translated into higher population densities in the following year (Staley and others 2007). Overall, our results suggest that medium-term climatic conditions primarily affect process rates through animal abundances directly or through vegetation effects, but the effect direction depends on the animal groups involved in each ecosystem process and in each habitat.

#### Short-Term Versus Medium-Term Effects of Land-Use Intensity

Although we could not distinguish between short- and medium-term forest management, grassland management could be split into a short-term (management during the spring and summer months prior to the survey) and a medium-term (the preceding 2 years) components. The effects of land-use activities in the short-term were generally stronger than the medium term for all processes, except for seed removal. We found negative effects of mowing and fertilization intensities on the process rates in grasslands. Previous studies focusing on time periods of preceding 5–10 years support our findings and relate the effects to declines in population densities directly or indirectly through vegetation (Gossner and others 2014; Simons and others 2014; Frank and others 2017; Meyer and others 2019). The reason for the stronger effects of short-term land-use intensities was probably that the immediate actions were more correlated to population densities and activities of the animals involved in our 2-day surveys. Our findings about the negative effects of short- and medium-term grazing on predation, contrary to nonsignificant findings of longer-term grazing reported by Meyer and others (2019), may support this idea.

In the short term, we also found positive effects of two land-use components in grasslands, in accordance with previous findings concerning longer time periods: grazing had a positive effect on dung removal (Frank and others 2017) and fertilization had a positive effect on predation by arthropods (Meyer and others 2019). Unlike mowing, grazing and fertilization improve resource availability for specific animal groups but do not directly kill them; grazing provides dung for dung beetles and fertilization provides nutrients for herbivores and their predators. In summary, the positive effects of land-use activities were likely facilitated by increased resources, and the negative effects resulted from the direct loss of individuals and changes to plant community structure and quality.

### Differences in Responses Between Grasslands and Forests

The effects of each explanatory variable on the process rates differed between forests and grasslands in terms of strength and direction. Furthermore, the driver sets (climatic vs. land-use or short-term vs. medium-term) did not have consistently higher or lower overall effect sizes in both habitats. These habitats differ substantially in terms of biomass, vegetation structure, and species composition; therefore, those differences are expected to be reflected in the animal-mediated process rates. Most prominently, climatic conditions had a positive effect on seed removal in grasslands but a negative effect in forests; this result could occur when a process is mediated by different animal groups in different habitats. For example, 2017 had high rodent activity in forests (Christian Imholt, pers. comm.), and we recorded higher rates of predation by rodents and seed removal in forests compared to grasslands. Therefore, we propose that rodents may have been involved in seed removal more in forests than in grasslands, thus causing some of the differences in the response of the seed removal rates to the climatic conditions in two habitats. Further studies on seed removal would benefit from experimental exclosures or remote cameras to determine the animal groups involved and their contribution to seed removal rates (Tschumi and others 2018).

The process rates were higher in forests compared to grasslands. This is probably due to higher biomass, abundance, and diversity of animals such as birds (Hurlbert 2004) and dung beetles (Frank and others 2017) resulting from higher primary productivity and habitat complexity in forests compared to grasslands (Evans and others 2005). Higher species richness and abundance in a habitat can result in higher process rates through several mechanisms. First, higher species richness provides a higher probability that a particularly influential species can be involved in the process (sampling effect, Loreau and others 2001). Second, different species may exploit a resource at different times of day or in different microhabitats (species complementarity or additive effects; Letourneau and others 2009). Third, higher richness ensures that each functional group and associated processes are maintained under heterogeneous or disruptive conditions due to varying responses of species within functional groups (redundancy or insurance model; Naeem and Li 1997). Finally, higher abundance results in higher rates because more individuals are involved in the process over time (mass effects, for example, Ebeling and others 2014).

## Conclusions

Two determinants of global change, land-use intensity and climatic conditions, had significant effects on the rates of animal-mediated ecosystem processes at short- and medium-term timescales. This supports the view that global change will not only impact animal diversity but also affect ecosystem functioning. Our findings on the effects of climatic versus land-use drivers suggest that none of the driver sets is more or less important overall; each driver had significant effects on animal-mediated processes, but their effects varied across habitats and processes.

Habitat type and vegetation structure were important in driving process rates in a temperate terrestrial environment. Recent land conversions, such as afforestation to mitigate climate change or vegetation clearings to restore semi-natural grasslands (Burrascano and others 2016), are therefore expected to have substantial effects on animal-mediated ecosystem processes. Human interventions that influence climatic conditions and land-use intensity are also expected to have an impact on animal-mediated ecosystem processes; however, our results show that effects will be process-specific and will vary between habitat types. This implies that there is no single optimal management strategy that consistently promotes all processes across habitats. Our findings provide hints about how to promote beneficial processes in different habitats, such as predation for pest control, seed removal, and dung removal as components of decomposition. Thus, our results provide a baseline for decision making of stakeholders and practitioners.

## Electronic supplementary material

Below is the link to the electronic supplementary material.

Supplementary material 1 (PDF 2304 kb)

## Data Availability

The data will become publicly available at https://www.bexis.uni-jena.de/ according to the Rules of Procedure of the German Science Foundation (DFG)-funded Biodiversity Exploratories.
