# Supplementary material for: Animal-Mediated Ecosystem Process Rates in Forests and Grasslands are Affected by Climatic Conditions and Land-Use Intensity
Source: Ecosystems. 2020 Aug 6;24(2):467–83. doi: 10.1007/s10021-020-00530-7 (PMC8550575; doi:10.1007/s10021-020-00530-7)
Supplement: Supplementary file 1 — Supplementary material 1 (PDF 2304 kb) [file 10021_2020_530_MOESM1_ESM.pdf]

Supplementary material for Ambarlı et al. "Animal-mediated ecosystem process rates in forests and grasslands are affected by climatic conditions and land-use intensity"

Authors: Didem Ambarlı*, Nadja K. Simons, Katja Wehner, Wiebke Kämper, Martin M. Gossner, Thomas Nauss, Felix Neff, Sebastian Seibold, Wolfgang Weisser, Nico Blüthgen

*Corresponding author; e-mail: didem.ambarli@gmail.com, phone: +49.8161.71.4861

Table of contents

S1: Relation of seed removal rate with short-term precipitation

S2: Details of herbivory measurements

S3: Details of processing explanatory data

S4: GLMM results in detail

S5: Comparison of the mean effect sizes of short-term vs. medium-term land-use variables in grasslands

S6: Process rates in detail

S7: Effects of the vegetation structure on process rates

S8: Linear correlations between the process rates

S9: Within-plot variation of the process rates

SUPPLEMENTARY MATERIAL S1 Relation of seed removal rate with short-term precipitation

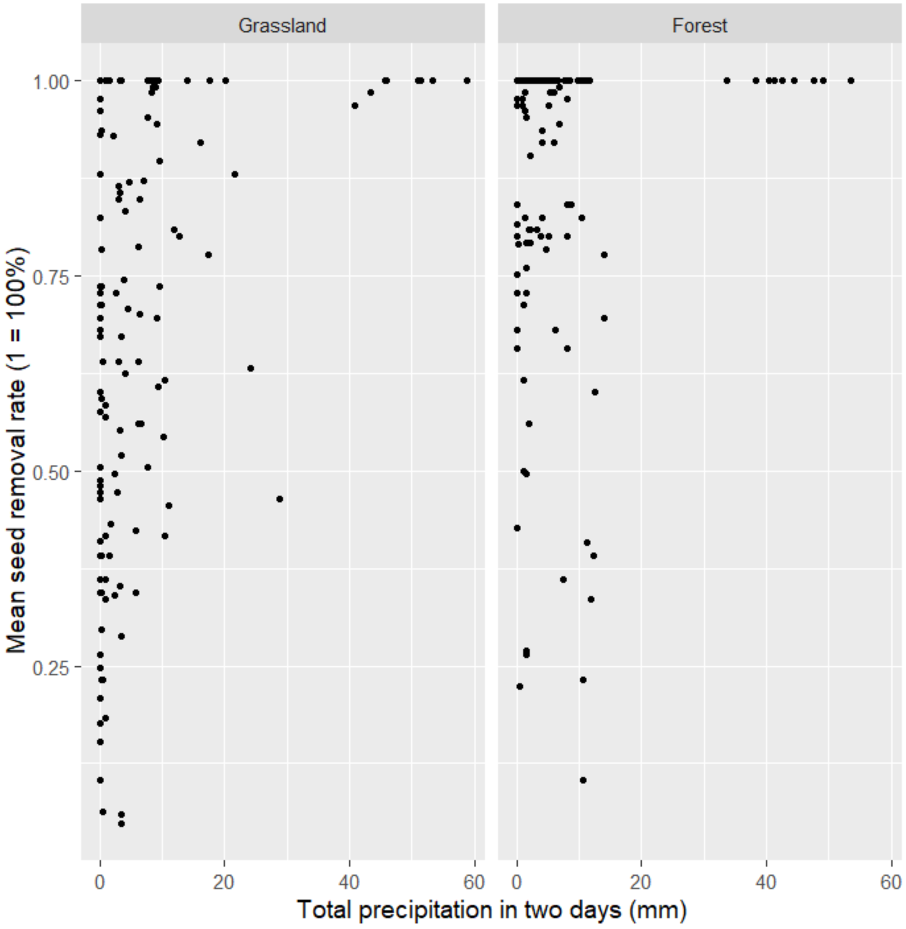

Figure S1 Scatterplot of mean seed removal rate per plot (original data) versus total precipitation in the survey periods in grasslands and forests. Complete removal after 30 mm precipitation implies removal due to wash-away. Therefore data from plots with higher than 30 mm total precipitation were excluded from the analyses.

SUPPLEMENTARY MATERIAL S2 Details of herbivory measurements

A. Quantifying herbivory in grasslands

In grasslands, herbivory assessments were based on biomass samples that were taken from a temporarily fenced area where early vertebrate grazing and mowing were excluded. A metal frame of 10 cm × 45 cm × 2 cm was placed randomly at two sampling locations in the fenced area. The vegetation was cut at the edge of the frame above 2 cm. In the lab, the proportion of grasses and stems or leaves of herbaceous plants were estimated visually to account for differences in herbivory between plant functional groups. Then an overall subset of 100 leaves was randomly picked from the two groups (herbs and grasses) according to their proportion in the biomass. Leaf damage was estimated as damaged surface area of each leaf in mm^2 by comparing the damaged areas in the leaves with circular and square templates ranging in size from 1 to 500 mm^2 (see Gossner and others, 2014). For each leaf, the total damaged area from different damage types (sucking, scraping, mining, chewing etc.) were summed per leave to get one value for damaged area per leaf. We reported regularly shaped, whitish points, which were often clearly visible against the light, as sucking damage caused by sap-sucking insects. Though this method may fail to include all sucking damage especially by small auchenorrhynchans, it captures the majority and is therefore used commonly in insect herbivory studies (Loranger and others, 2014). Leaf damage at the edges was estimated by reconstructing the removed area based on leaf shape. The remaining leaf area was measured with a leaf-area-meter (LI-COR area meter (LI-3100C, Lincoln (NE) USA)). Herbivory rates were calculated as the total damaged area divided by the total leaf area, i.e. damaged and remaining. We accounted for the damage types also measured by leaf-area-meter so included in the remaining area, i.e. sucking, scraping, mining and galling, by subtracting the total area of those damages from the measured area to find the remaining area.

B. Quantifying herbivory

In forests, herbivory rate was estimated on the 10 (in ALB and HAI regions) and 8 (in SCH region) most abundant plant species of all forest layers in terms of percent cover. This also included geophyte species sampled in spring, which were excluded from the subsequent analyses to enable use of the climatic data in the short-term timescale. We selected those species based on plant survey data from former years (Boch and others, 2013, D.Prati, pers. comm). The leaf material was collected along the outer border of the plots in summer (from mid-June (ALB) to mid-August (SCH)). Leaves from the tree layer were collected using elongated lopping shears (max. height: 7 m) to cut branches from the shaded canopy of at least three trees. Where shears were not long enough, branches were collected using shooting and/or climbing. When individuals of the tree species were present in the shrub layer, leaves were collected from that layer as well in an amount approximately representing the distribution of tree and shrub layer within the plot. When a species could not be found on the plot margin or when sampling was not possible out of other reasons, this species was replaced by the next abundant species on the species list. All plant material was collected in plastic bags containing a moist cloth to prevent the leaves from drying out and transferred to a fridge until further processing. In the lab, leaf material was further processed. A total of 12 to 200 randomly picked leaves per species per plot were processed (depending on leaf size and composition, TableS1). For each single leaf, the area was measured using a LI-COR area meter (LI-3000C, Lincoln (NE) USA). For coniferous trees (*Abies*, *Picea*, *Pinus* sp.), sets of 10 leaves were measured together and average values were taken for a single leaf area for better accuracy. For grass species with needle-shaped leaves (*Deschampsia flexuosa*), no reliable area measurements were possible using the leaf area meter. Thus, leaf length was measured using a ruler and the approximate area was determined by multiplying the length by an estimated leaf width of 1 mm. As for grassland herbivory, the total damaged area from different damage types (sucking, scraping, mining, chewing, etc.) was determined for each leaf using circular and square templates ranging in size from 1 to 500 mm² (see Gossner and others, 2014). Damage types were summed and herbivory rate per leaf was calculated as the proportion of herbivory-affected leaf

area compared to the corrected total area of the leaf (i.e., the sum of measured leaf area and chewing damage). From those leaf-level herbivory rates, we calculated mean herbivory rates per plant species and plot. To determine community-level herbivory rates per plot, we used plant cover values obtained from plot core areas by vegetation surveys, the same data as used for the selection of the most abundant plant species. Plant-species herbivory rates were weighted by their relative contribution to the total cover of assessed plants to calculate community-level herbivory rates per plot.

Table S1 Number of leaves measured for different taxa in forest plots

Number of leaves	Sampled plant species
12	*Pteridium aquilinum*
20	*Actaea spicata, Aegopodium podagraria, Aesculus hippocastanum, Allium ursinum, Arum maculatum, Athyrium filix-femina, Cardamine bulbifera, Dryopteris carthusiana, Dryopteris dilatata, Dryopteris filix-mas, Fraxinus excelsior, Gymnocarpium dryopteris, Mercurialis perennis* (part.), *Paris quadrifolia, Robinia pseudoacacia, Sorbus aucuparia*
40	*Calamagrostis epigejos*
50	*Rubus fruticosus corylifolius* agg., *Rubus idaeus, Sambucus nigra, Sambucus racemosa*
80	*Agrostis capillaris, Agrostis stolonifera, Anthoxanthum odoratum, Brachypodium pinnatum, Brachypodium sylvaticum, Calamagrostis arundinacea, Carex montana, Carex pilulifera, Carex remota, Carex sylvatica, Dactylis glomerata, Dactylis polygama, Deschampsia cespitosa, Deschampsia flexuosa, Festuca gigantea, Hordelymus europaeus, Juncus effusus, Melica uniflora, Milium effusum, Poa chaixii, Poa nemoralis, Poa trivialis, Stellaria holostea*
100	*Acer campestre, Acer platanoides, Acer pseudoplatanus, Alliaria petiolata, Anemone nemorosa, Anemone ranunculoides, Asarum europaeum, Betula pendula, Carpinus betulus, Circaea lutetiana* agg., *Conyza canadensis, Corylus avellana, Crataegus* sp., *Euphorbia amygdaloides, Fagus sylvatica, Fragaria vesca, Frangula alnus, Galeopsis pubescens, Galium aparine, Galium odoratum, Galium rotundifolium, Galium sylvaticum, Geranium robertianum, Hedera helix, Hypericum perforatum, Impatiens noli-tangere, Impatiens parviflora, Lamium galeobdolon* agg., *Lonicera xylosteum, Melampyrum pratense, Mercurialis perennis* (part.), *Moehringia trinervia, Mycelis muralis, Oxalis acetosella, Polygonatum multiflorum, Polygonatum verticillatum, Populus tremula, Primula elatior veris* agg., *Prunella vulgaris, Prunus avium, Prunus serotina, Prunus spinosa, Quercus* sp., *Ranunculus ficaria, Ranunculus repens, Rumex acetosella, Salix caprea, Sanicula europaea, Senecio ovatus, Stachys sylvatica, Tilia cordata, Tilia europaea, Tilia platyphyllos, Ulmus glabra, Ulmus laevis, Urtica dioica, Vaccinium myrtillus, Veronica officinalis, Viola reichenbachiana riviniana* agg.
200	*Abies alba, Picea abies, Pinus sylvestris*

SUPPLEMENTARY MATERIAL S3 Details of processing explanatory data

A. Descriptions

To quantify short-term climatic conditions, we used precipitation sum and the mean or maximum of daily temperature (°C) of the survey period, depending on the process in question. To quantify medium-term effects, we focused on warm and cold periods separately. For the warm period (springs and summers) in 2016 and 2017, we used precipitation sum, mean of the maximum daily temperature reflecting warm conditions, and number of cool days, i.e. with maximum temperature < 10°C of air temperature. For the cold period, we used the minimum of the daily minimum temperature of September and overall winter. The start and end dates of those periods were as follows: 26 March to 19 Sept 2016 for warm season of 2016, 20 Sept 2016 to 28 March 2017 for cold season and from 29 March to starting date of the surveys for warm season of 2017 for each plot. We set those days based on the start or end of successive frost days (a day with an hourly temperature below 0°C). This approach is similar to the general April/September division but more detailed on a daily basis.

Table S2 Description of the climatic variables

Name	Abbreviation	Description	Unit
Cool days	CoolD	Days with maximum temperature < 10°C of air temperature	d (day)
Maximum temperature	TempMax	Maximum of daily temperature of the air at 2 meters above ground	°C
Mean temperature	TempMean	Mean of daily temperature of the air at 2 meters above ground	°C
Precipitation	Prec	Daily Precipitation based on RADOLAN product	mm
Minimum temperature in September	September_Tmin	Minimum of air temperature recorded in September 2016	°C
Minimum temperature in winter	Winter_Tmin	Minimum of air temperature recorded in winter 2016	°C

Climate data were measured by climate stations installed at each plot (https://www.bexis.uni-jena.de/). When data were missing for specific dates of a plot, we substituted it with the mean value of the specific habitat of the region for that date. Other parameters that might affect the processes rates, specifically mean wind velocity and sunshine duration, were not used because of the high number of missing data in

the dataset. We did not use the number of frost days in summer periods, as in each summer, there was only one plot with frost days.

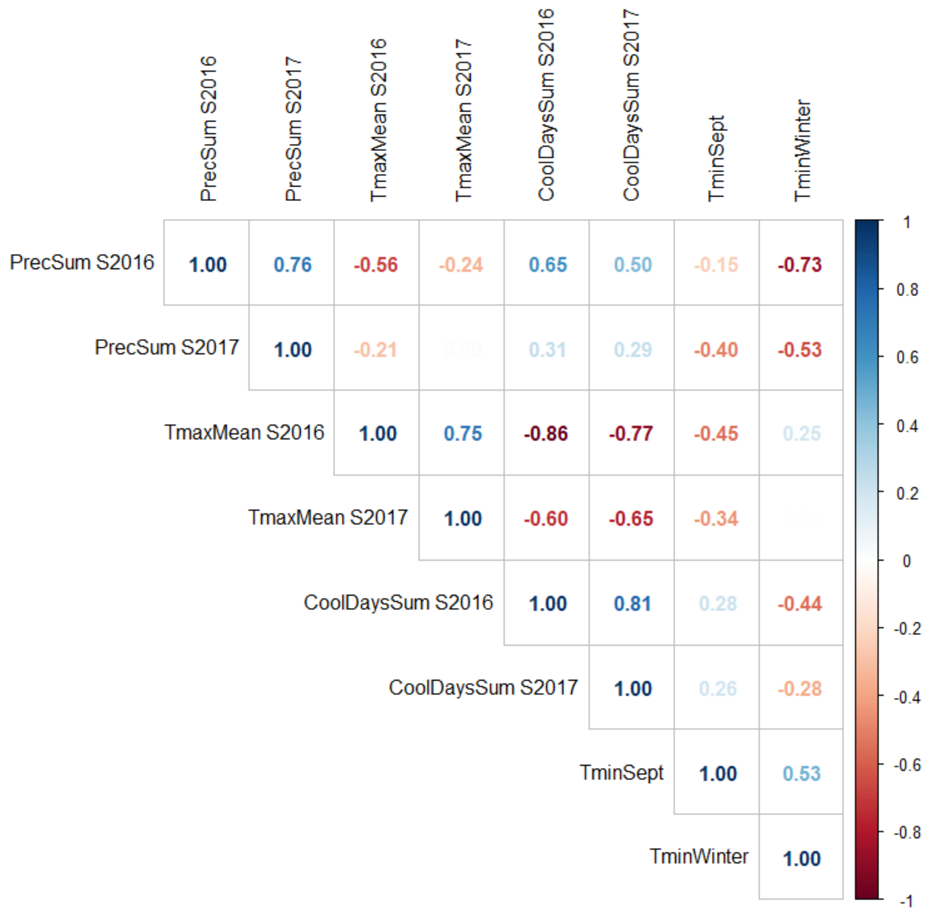

Figure S2 Correlations between the medium-term climatic variables used in the dung removal, seed removal and predation analyses. Numbers indicate Pearson correlation coefficients. Blue color indicates a positive correlation whereas red color indicates a negative correlation. Correlations smaller than 0.01 appear white. PrecSum: Precipitation sum, TmaxMean: Mean of the maximum temperature, CoolDaysSum: Total number of cool days for the indicated period, S2016: spring+summer 2016, S2017: spring+summer 2017. TminSept and TminWinter are minimum temperatures for September 2016 and winter 2016, respectively.

B. Principal component analysis (PCA) of climatic data

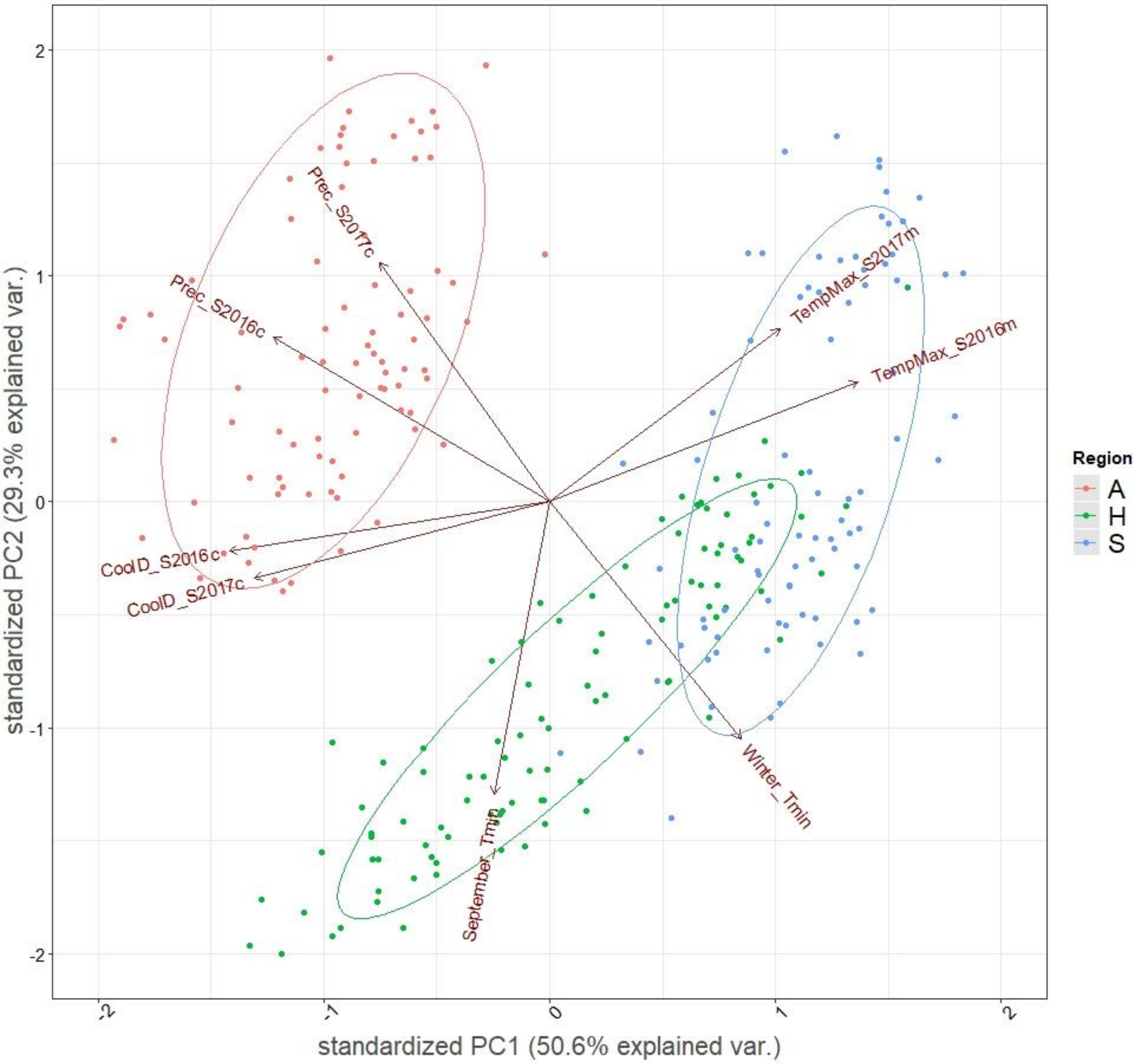

Figure S3 Ordination of the first two axis of PCA on medium-term climatic variables used in the dung removal, seed removal and predation analyses. PCA was conducted with "factoextra" package (Kassambara and Mundt, 2017) and plotted with ggbiplot package in R. Tmax: Maximum temperature, CoolD: number of cool days in that period, Prec: Precipitation. S2016 and S2017 indicate variables for spring and summer seasons of 2016 and 2017, respectively. September_Tmin and Wnt_Tmin indicate minimum temperatures in September 2016 and across winter between 2016 and 2017, respectively. Letters "c" and "m" at the end of each variable indicate cumulative or mean of that variable for that period, respectively. Plots of each region are colored and indicated in the legend as A: Schwäbische Alb in red, H: Hainich-Dün in green, and S: Schorfheide-Chorin in blue.

Table S3 Contributions of the medium-term climatic variables to dimensions of the PCA in Fig S3 above. Variables are sorted according to the contribution to the first dimension. S2016: spring and summer of 2016, S2017: spring and summer of 2017

**Medium-term climatic variables**	**Dim.1**	**Dim.2**	**Dim.3**	**Dim.4**	**Dim.5**	**Dim.6**	**Dim.7**	**Dim.8**
Number of cool days in S2016	21.3	0.9	0.0	5.9	11.9	26.5	29.4	4.1
Mean of maximum temperatures of S2016	19.7	5.2	0.2	0.9	5.2	20.2	39.2	9.4
Number of cool days in S2017	18.1	2.1	0.1	18.5	40.0	16.1	2.3	2.9
Precipitation sum of S2016	15.9	9.7	5.8	4.9	10.2	12.4	12.1	29.1
Mean of maximum temperatures of S2017	11.0	10.8	25.5	10.8	28.0	2.6	4.8	6.5
Minimum temperature of winter 2016	7.6	20.1	15.4	23.8	0.9	6.5	10.7	15.0
Precipitation sum of S2017	6.0	20.5	26.0	22.0	3.3	1.3	0.9	20.1
Minimum temperature of September 2016	0.6	30.8	27.0	13.2	0.5	14.4	0.5	13.0
**Cumulative explained variance**	50.6	79.8	87.9	92.8	96.6	98.2	99.2	100

Table S4 Correlations of the medium-term climatic variables to dimensions of the PCA in Fig S3. Variables are sorted according to the correlation coefficients for the first dimension. S2016: spring and summer of 2016, S2017: spring and summer of 2017

**Medium-term climatic variables**	**Dim.1**	**Dim.2**	**Dim.3**	**Dim.4**	**Dim.5**	**Dim.6**	**Dim.7**	**Dim.8**
Mean of maximum temperatures of S2016	0.89	0.35	0.04	-0.06	0.13	0.16	-0.18	-0.08
Mean of maximum temperatures of S2017	0.67	0.50	-0.40	0.21	0.29	-0.06	0.06	0.06
Minimum temperature of winter 2016	0.55	-0.69	-0.31	-0.31	-0.05	-0.09	-0.09	0.10
Minimum temperature of September 2016	-0.16	-0.85	-0.42	0.23	-0.04	0.14	0.02	-0.09
Precipitation sum of S2017	-0.49	0.69	-0.41	-0.30	-0.10	-0.04	0.03	-0.11
Precipitation sum of S2016	-0.80	0.48	-0.19	0.14	-0.18	0.13	-0.10	0.14
Number of cool days in S2017	-0.86	-0.22	0.03	-0.27	0.35	0.14	0.04	0.04
Number of cool days in S2016	-0.93	-0.14	-0.01	0.15	0.19	-0.18	-0.15	-0.05

Table S5 Descriptive statistics for the climatic variables used in the dung removal, seed removal and predation analyses (temperature in °C and precipitation in mm). Note: Surveys took place over a 48 h period.

**Variables**	**Min.**	**1st Qu.**	**Median**	**Mean**	**3rd Qu.**	**Max.**
Precipitation sum of survey period	0.0	0.8	3.5	7.2	8.2	58.9
Mean temperature of survey period	12.6	15.8	17.1	17.4	18.6	24.8
Max temperature of survey period	13.5	17.6	18.9	19.1	20.6	25.7
Precipitation sum of spring+summer 2016	188.5	250.4	325.7	424.1	653.7	806.2
Precipitation sum of spring+summer 2017	68.0	95.2	127.6	132.5	169.5	226.6
Mean of maximum daily temperatures in spring+summer 2016	16.4	18.2	19.8	19.7	20.7	24.3
Mean of maximum daily temperatures in spring+summer 2017	13.7	16.1	16.8	16.8	17.6	19.7
Number of cool days in spring+summer 2016	2	7	13	10.9	14	28
Number of cool days in spring+summer 2017	4.0	9.0	12.0	11.9	14	23
Minimum temperature in September 2016	-1.5	3.2	5.1	4.6	6.53	8.8
Minimum temperature in winter	-25.9	-15.5	-14.2	-14.6	-12.2	-9.5

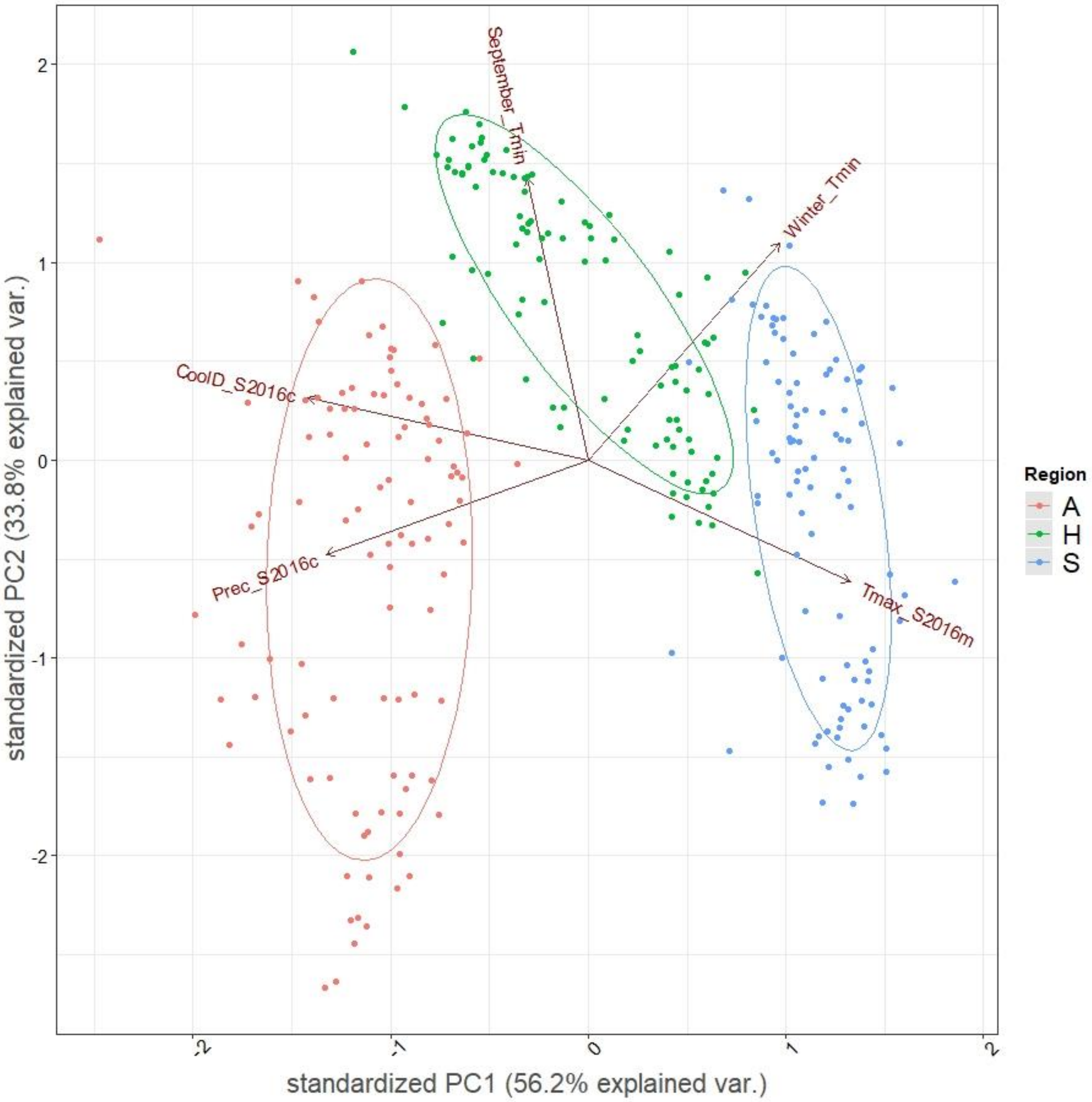

Figure S4 Ordination of the first two axis of PCA on medium-term climatic variables used in the herbivory analyses. It was conducted with "factoextra" package (Kassambara and Mundt, 2017) and plotted with ggbiplot package (Vu, 2011) in R. Tmax: Maximum temperature, CoolD: number of cool days in that period, Prec: Precipitation. S2016 indicates variables for the spring and summer of 2016. September_Tmin and Wnt_Tmin indicate minimum temperatures in September 2016 and across winter between 2016 and 2017, respectively. Letters "c" and "m" at the end of each variable indicate cumulative or mean of that variable for that period, respectively. Plots of each region are colored and indicated in the legend as A: Schwäbische Alb in red, H: Hainich-Dün in green, and S: Schorfheide-Chorin in blue.

Table S6 Contributions of the medium-term climatic variables to dimensions of the PCA for the herbivory analyses in Fig S4. Variables are sorted according to the contribution to the first dimension. S2016 indicates spring and summer of 2016.

**Medium-term climatic variables**	**Dim.1**	**Dim.2**	**Dim.3**	**Dim.4**	**Dim.5**
Number of cool days in S2016	30.6	2.5	19.6	0.6	46.7
Mean of maximum temperatures of S2016	26.8	9.6	9.0	13.4	41.3
Precipitation sum of S2016	26.8	5.8	53.9	11.3	2.3
Minimum temperature of winter 2016	14.4	30.5	0.6	45.1	9.4
Minimum temperature of September 2016	1.4	51.6	16.9	29.7	0.4
**Cumulative explained variance**	56.2	89.9	94.8	97.9	100

Table S7 Correlations of the medium-term climatic variables to dimensions of the PCA for the herbivory analysis in Fig S4. Variables are sorted according to the correlation coefficients for the first dimension. S2016 indicates spring and summer of 2016.

**Medium-term climatic variables**	**Dim.1**	**Dim.2**	**Dim.3**	**Dim.4**	**Dim.5**
Number of cool days in S2016	-0.93	0.2	-0.22	-0.03	0.22
Precipitation sum of S2016	-0.87	-0.31	0.36	0.13	0.05
Minimum temperature of September 2016	-0.2	0.93	0.2	-0.21	-0.02
Minimum temperature of winter 2016	0.64	0.72	0.04	0.26	0.1
Mean of maximum temperatures of S2016	0.87	-0.4	0.15	-0.14	0.21

C. Histograms of the climate variables

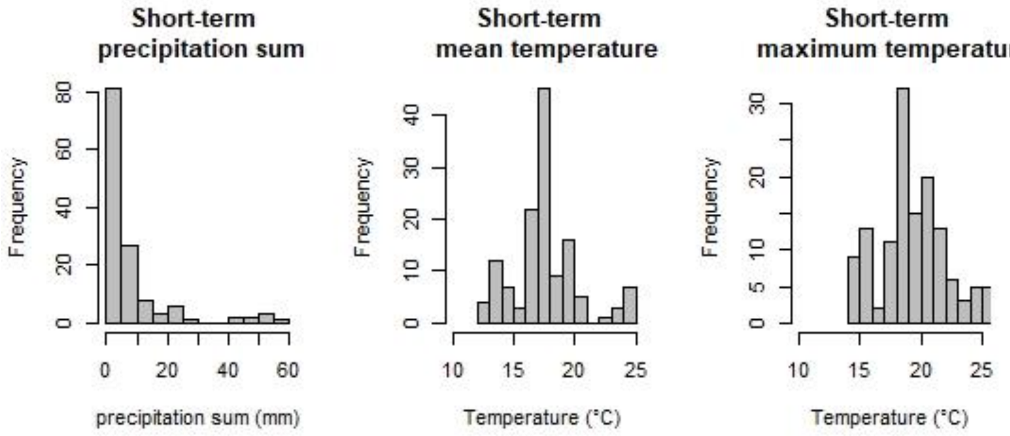

Figure S5 Histogram of the climatic conditions during the short-term timescale in grasslands (used for the analyses of the dung removal, seed removal and predation)

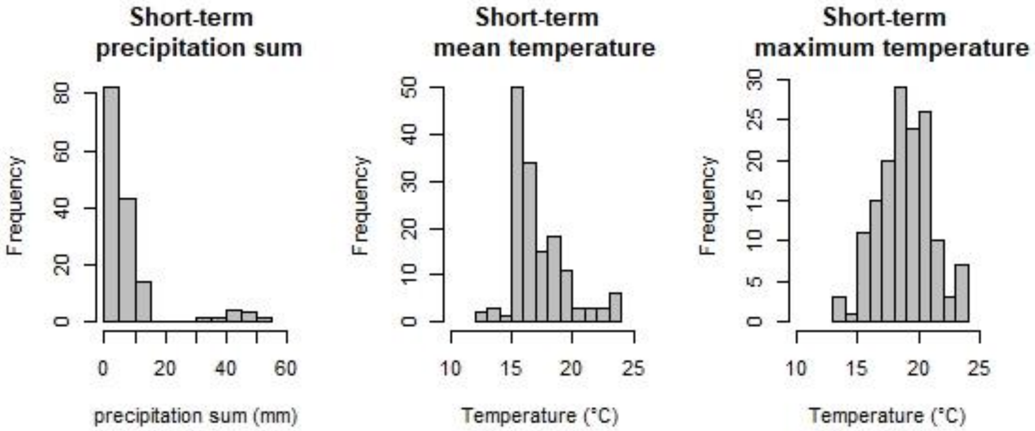

Figure S6 Histogram of the climatic conditions during the short-term timescale in forests (used for the analyses of the dung removal, seed removal and predation)

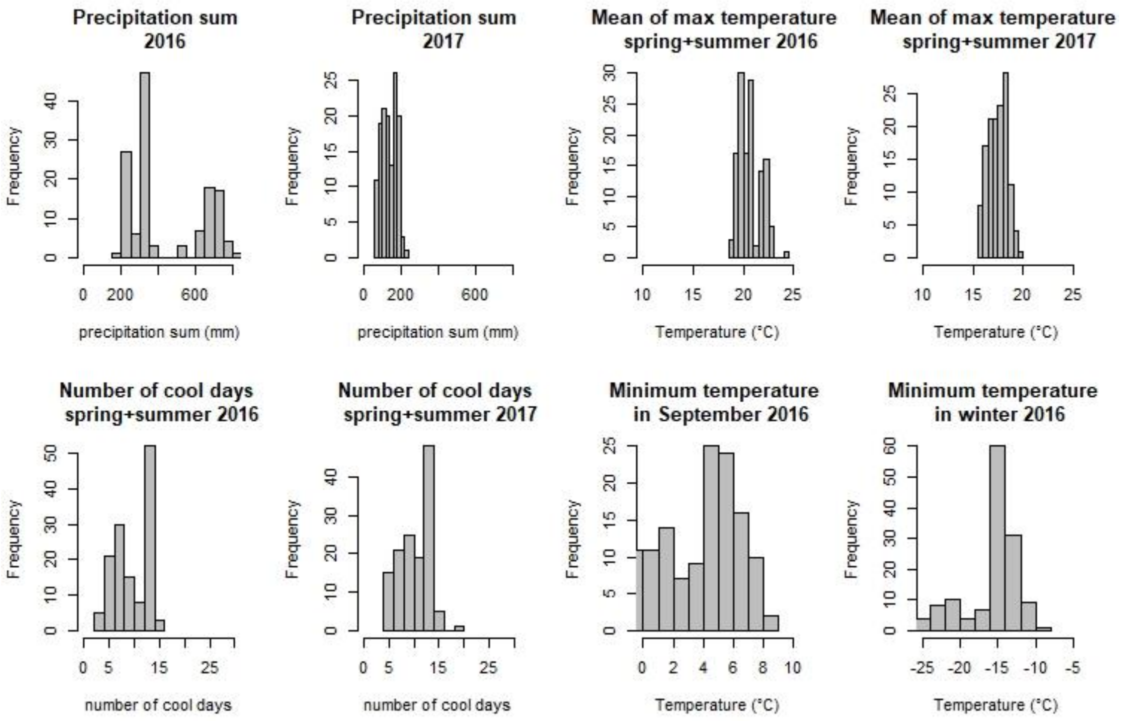

Figure S7 Histogram of the climatic conditions during the medium-term timescale in grasslands (used for the analyses of the dung removal, seed removal and predation)

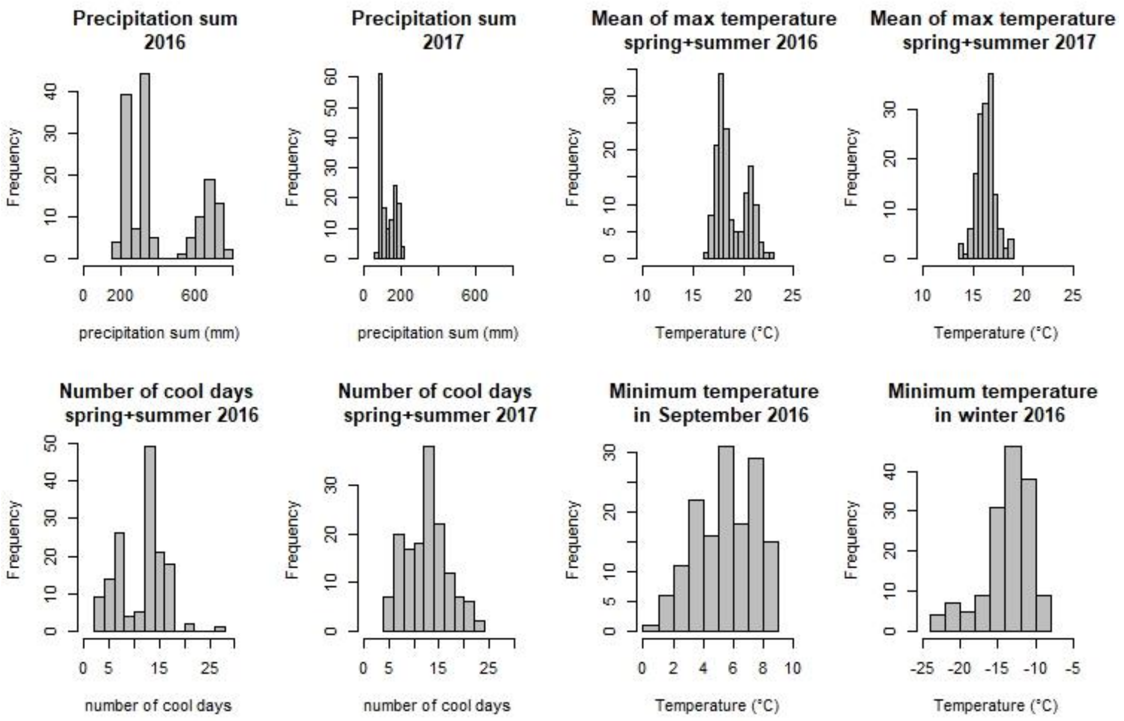

Figure S8 Histogram of the climatic conditions during the medium-term timescale in forests (used for the analyses of the dung removal, seed removal and predation)

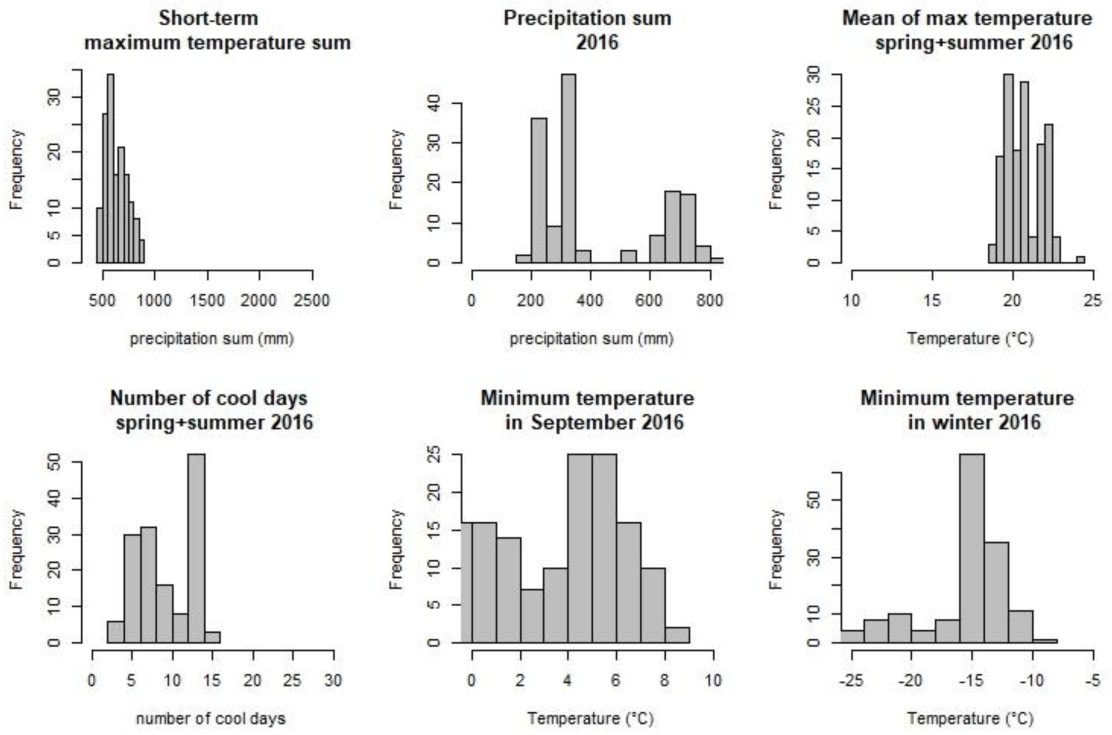

Figure S9 Histograms of the climate variables used for the herbivory in grasslands

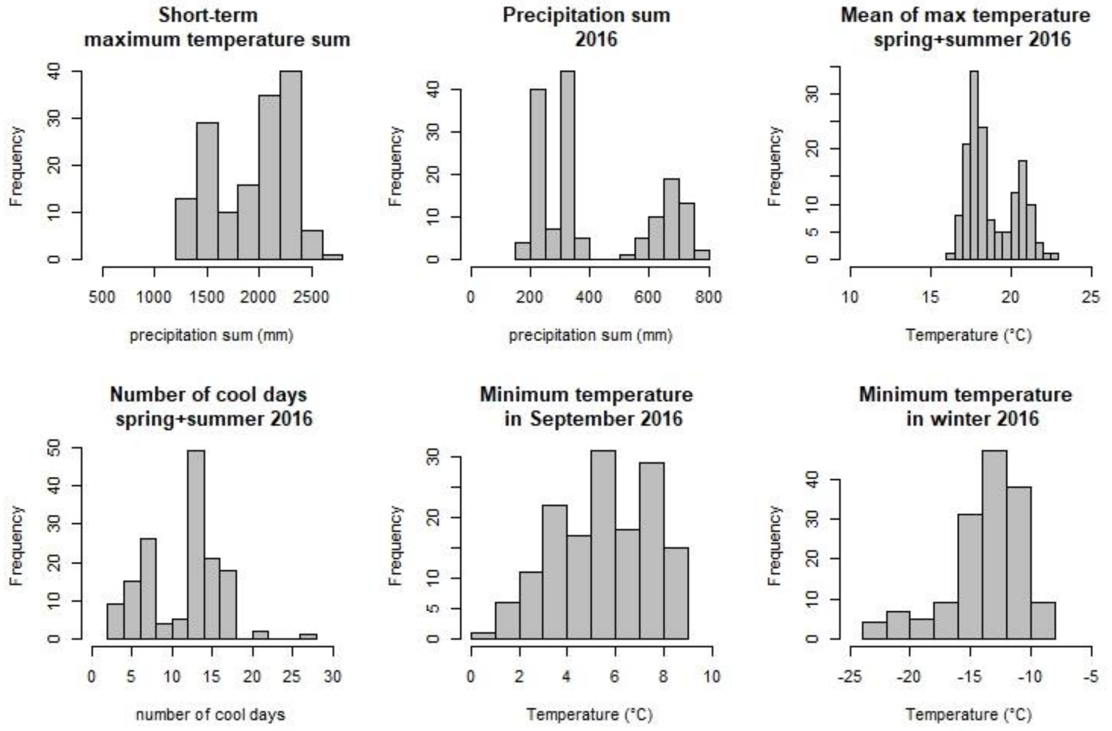

Figure S10 Histograms of the climate variables used for herbivory in forests

D. Coefficient of variation (CV) of the climate variables

Table S8 CV of climate variables that were used in the analyses of the dung removal, seed removal and predation

Habitat	Timescale	Variable	CV %
Forest	Short-term	Precipitation sum	154
Forest	Short-term	Temperature - mean	13
Forest	Short-term	Temperature – max	11
Forest	Medium-term	Precipitation sum for spring+summer 2016	46
Forest	Medium-term	Precipitation sum for spring+summer 2017	32
Forest	Medium-term	Mean of maximum temperature for spring+summer 2016	8
Forest	Medium-term	Mean of maximum temperature for spring+summer 2017	6
Forest	Medium-term	Number of cool days in spring+summer 2016	39
Forest	Medium-term	Number of cool days in spring+summer 2017	32
Forest	Medium-term	Minimum temperature in September 2016	36
Forest	Medium-term	Minimum temperature in winter 2016	-22
Grassland	Short-term	Precipitation sum	162
Grassland	Short-term	Temperature - mean	15
Grassland	Short-term	Temperature – max	14
Grassland	Medium-term	Precipitation sum for spring+summer 2016	46
Grassland	Medium-term	Precipitation sum for spring+summer 2017	29
Grassland	Medium-term	Mean of maximum temperature for spring+summer 2016	5
Grassland	Medium-term	Mean of maximum temperature for spring+summer 2017	5
Grassland	Medium-term	Number of cool days in spring+summer 2016	35
Grassland	Medium-term	Number of cool days in spring+summer 2017	27
Grassland	Medium-term	Minimum temperature in September 2016	70
Grassland	Medium-term	Minimum temperature in winter 2016	-23

Table S9 CV of climate variables that were used in the analyses of the herbivory

Habitat	Timescale	Variable	CV %
Forest	Short-term	Sum of daily maximum temperature	18
Forest	Medium-term	Precipitation sum for spring+summer 2016	46
Forest	Medium-term	Mean of maximum temperature for spring+summer 2016	8
Forest	Medium-term	Number of cool days in spring+summer 2016	40
Forest	Medium-term	Minimum temperature in September 2016	36
Forest	Medium-term	Minimum temperature in winter 2016	-22
Grassland	Short-term	Sum of daily maximum temperature	17
Grassland	Medium-term	Precipitation sum for spring+summer 2016	48
Grassland	Medium-term	Mean of maximum temperature for spring+summer 2016	6
Grassland	Medium-term	Number of cool days in spring+summer 2016	37
Grassland	Medium-term	Minimum temperature in September 2016	77
Grassland	Medium-term	Minimum temperature in winter 2016	-22

E. Histograms of land-use variables

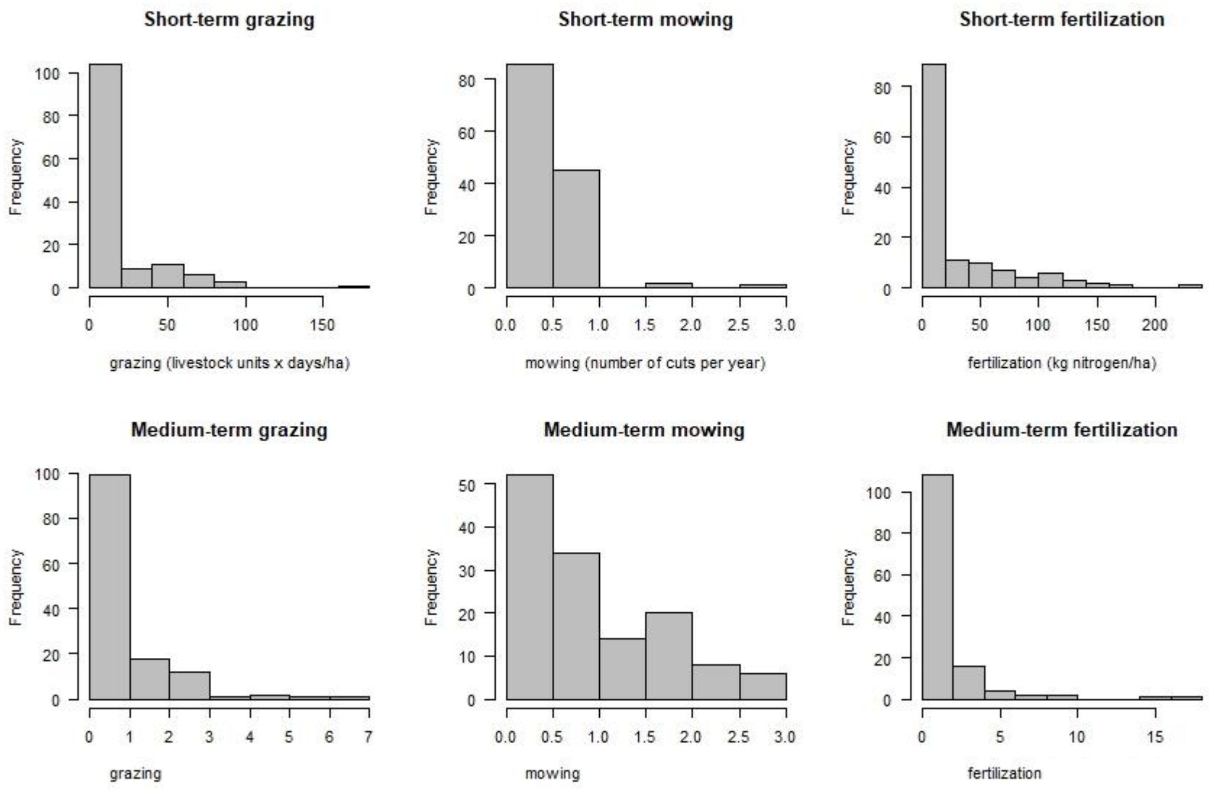

Figure S11 Histograms of the land-use data used in the dung removal, seed removal and predation analyses. The x-axes of the first row include variables at short-term grazing (livestock unit*days/ha), mowing (number of cuts) and fertilization (kg nitrogen/ha), respectively. The second row has the same variables for the medium-term timescale, standardized relative to its mean within that year, and then calculated the average of two preceding years (2015 and 2016).

F. Correlations among explanatory variables used in the analyses

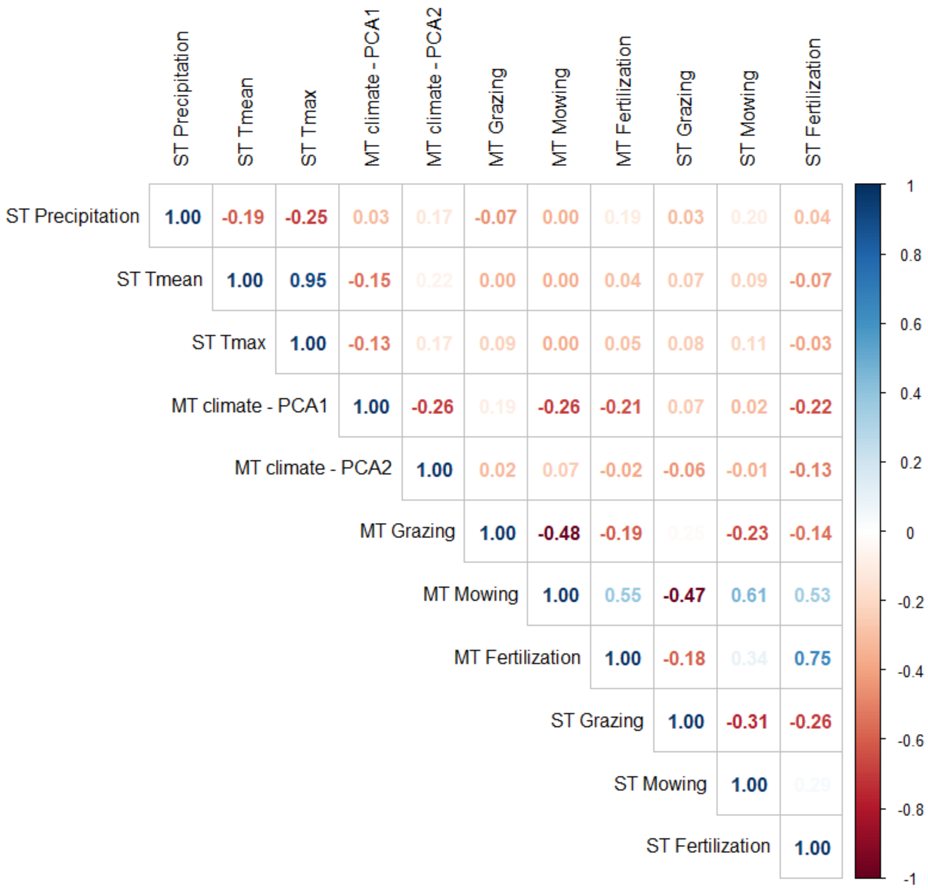

Figure S12 Correlations among grassland explanatory variables used for the dung removal, seed removal and predation analyses. Numbers indicate correlation coefficients based on Pearson's test. Blue color indicates a positive correlation whereas red color indicates a negative correlation. Positive correlations which are smaller than 0.25 appear white. ST: short-term variables corresponding to two days of survey period. MT: medium-term. MT variables are represented with the first two axes of the PCA. Short-term precipitation is the sum of daily precipitation of 2 days. Tmean: mean of daily temperature, Tmax: mean of daily maximum temperature.

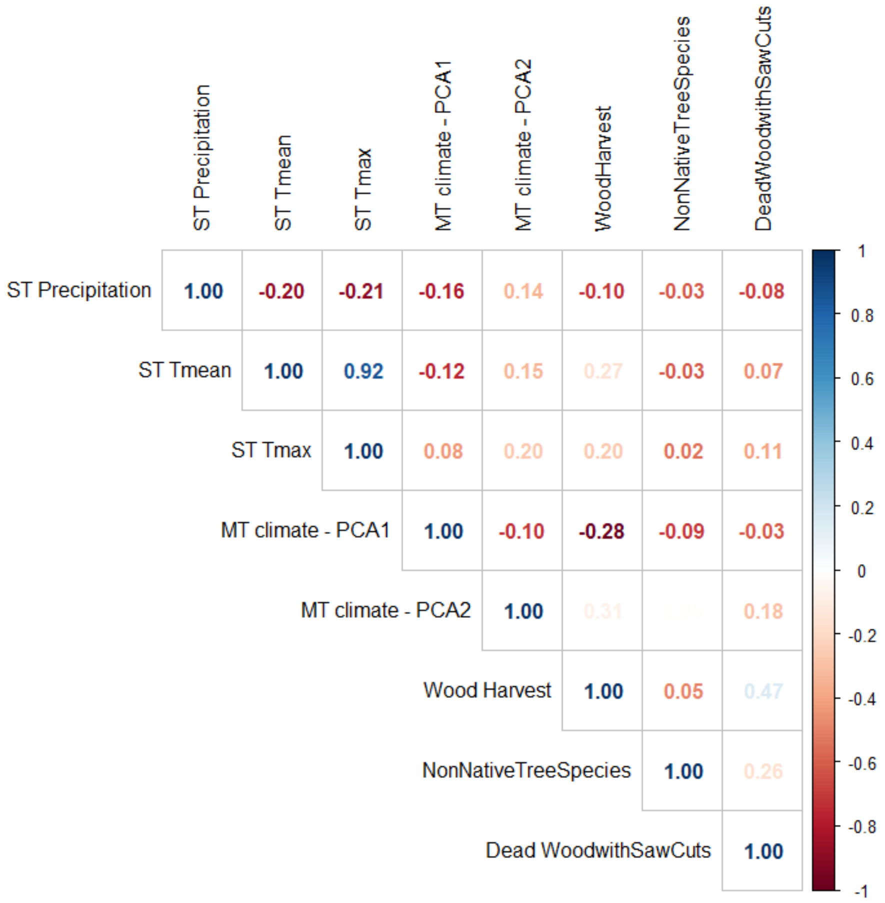

Figure S13 Correlations among forest explanatory variables used for dung removal, seed removal and predation analyses. Numbers indicate correlation coefficients based on Pearson test. Blue color indicates a positive correlation whereas red color indicates a negative correlation. Positive correlations smaller than 0.35 appear white. ST: short-term variables corresponding to two days of survey period. MT: medium-term. MT variables are represented with the first two axes of the PCA. Short-term precipitation is the sum of daily precipitation of 2 days. Tmean: mean temperature, Tmax: maximum temperature.

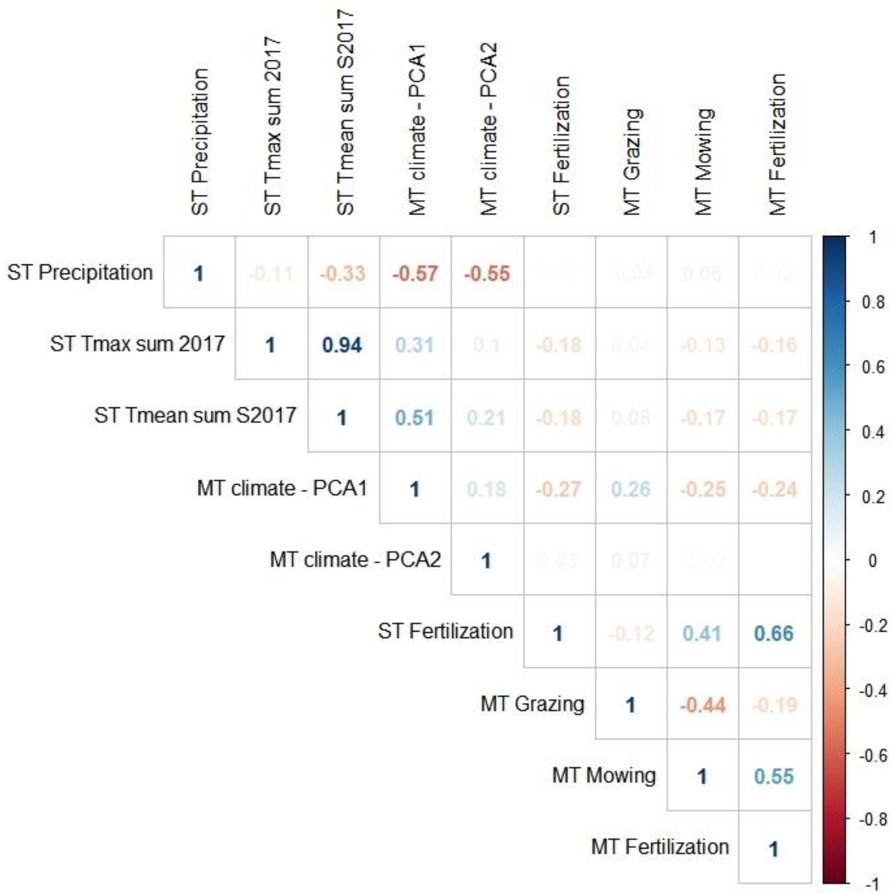

Figure S14 Correlations among grassland explanatory variables considered for the herbivory analyses. Numbers indicate correlation coefficients based on Pearson test. Blue color indicates a positive correlation whereas red color indicates a negative correlation. Correlations that are very close to zero appear white. ST: short-term variables corresponding to two days of survey period. MT: medium-term. MT variables are represented with the first two axes of the PCA. Precipitation is the sum of daily precipitation of the short-term period. Tmean: mean temperature, Tmax: maximum temperature. cum: cumulative.

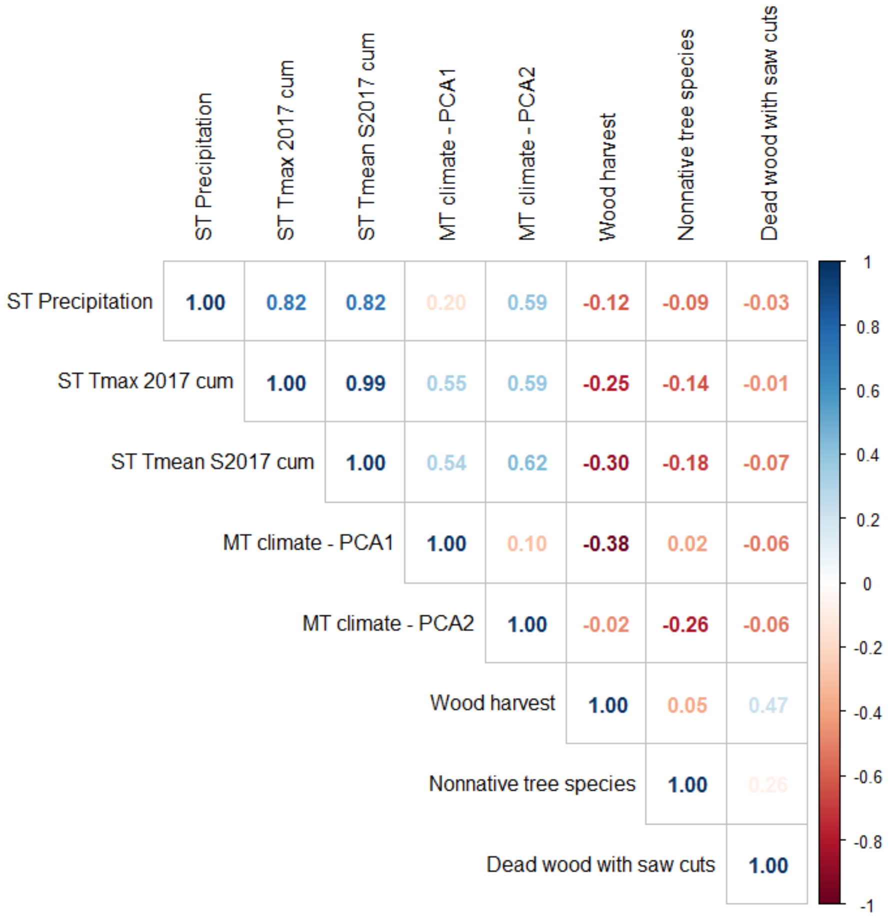

Figure S15 Correlations among forest explanatory variables considered for the herbivory analyses. Numbers indicate correlation coefficients based on Pearson test. Blue color indicates a positive correlation whereas red color indicates a negative correlation. ST: short-term variables corresponding to two days of survey period. MT: medium-term. MT variables are represented with the first two axes of the PCA. Precipitation is the sum of daily precipitation of the short-term period. Tmean: mean temperature, Tmax: maximum temperature, cum: sum of daily values over the period.

SUPPLEMENTARY MATERIAL S4 GLMM results in detail

Significance codes indicated in bold are as follows: (“.” : p≤0.1, *: p≤0.05,**: p≤0.01,***: p≤ 0.001.

A. Dung removal

Table S10 Dung removal in grasslands

**GLMM results (657 observations, 134 plots, 3 regions)**				
**Fixed effects***	**Estimate**	**Std.Error**	**t value**	**Pr(>|z|)**
(Intercept)	0.137	0.015	9.312	<0.001***
ST maximum temperature	0.031	0.018	1.788	0.074 .
ST precipitation	-0.016	0.019	-0.850	0.395
ST temperature: ST precipitation	-0.011	0.034	-0.317	0.751
**MT climate: Dry summer (PCA axis1)**	**0.031**	**0.014**	**2.295**	**0.022***
MT climate: Cold winter (PCA axis2)	-0.014	0.013	-1.094	0.274
MT DrySummer: ColdWinter	-0.016	0.015	-1.011	0.312
**ST grazing**	**0.026**	**0.013**	**2.093**	**0.036***
ST Mowing	0.014	0.013	1.119	0.263
ST Fertilization	-0.007	0.013	-0.584	0.559

ST: short-term, MT: medium-term

Table S11 Dung removal in forests

**GLMM results (723 observations, 146 plots, 3 regions)**				
**Fixed effects**	**Estimate**	**Std.Error**	**t value**	**Pr(>|z|)**
(Intercept)	0.249	0.093	2.679	0.007**
ST maximum temperature	-0.023	0.016	-1.427	0.154
**ST precipitation**	**0.039**	**0.019**	**2.041**	**0.041***
ST temperature: ST precipitation	-0.013	0.023	-0.569	0.569
MT climate: Dry summer (PCA axis1)	-0.047	0.047	-0.995	0.320
MT climate: Cold winter (PCA axis2)	0.018	0.033	0.563	0.574
MT DrySummer:ColdWinter	0.010	0.022	0.436	0.663
Wood harvest	-0.029	0.019	-1.547	0.122
**Non-natural trees**	**0.041**	**0.018**	**2.261**	**0.024***
**Anthropogenic deadwood**	**0.039**	**0.017**	**2.331**	**0.020***

ST: short-term, MT: medium-term

B. Seed removal

Table S12 Seed removal in grasslands

**GLMM results (657 observations, 134 plots; 3 regions)**				
**Fixed effects***	**Estimate**	**Std.Error**	**t value**	**Pr(>|z|)**
(Intercept)	1.662	0.236	7.043	<0.001***
ST maximum temperature	-0.008	0.247	-0.032	0.974
**ST precipitation**	**0.522**	**0.254**	**2.054**	**0.040***
ST temperature: ST precipitation	0.472	0.377	1.251	0.211
MT climate: Dry summer (PCA axis1)	0.304	0.244	1.247	0.212
**MT climate: Cold winter (PCA axis2)**	**0.749**	**0.216**	**3.472**	**<0.001*** **
**MT DrySummer: ColdWinter**	**0.919**	**0.258**	**3.564**	**<0.001*** **
ST grazing	-0.251	0.193	-1.301	0.193
ST Mowing	-0.205	0.203	-1.006	0.314
ST Fertilization	-0.106	0.196	-0.540	0.589

ST: short-term, MT: medium-term

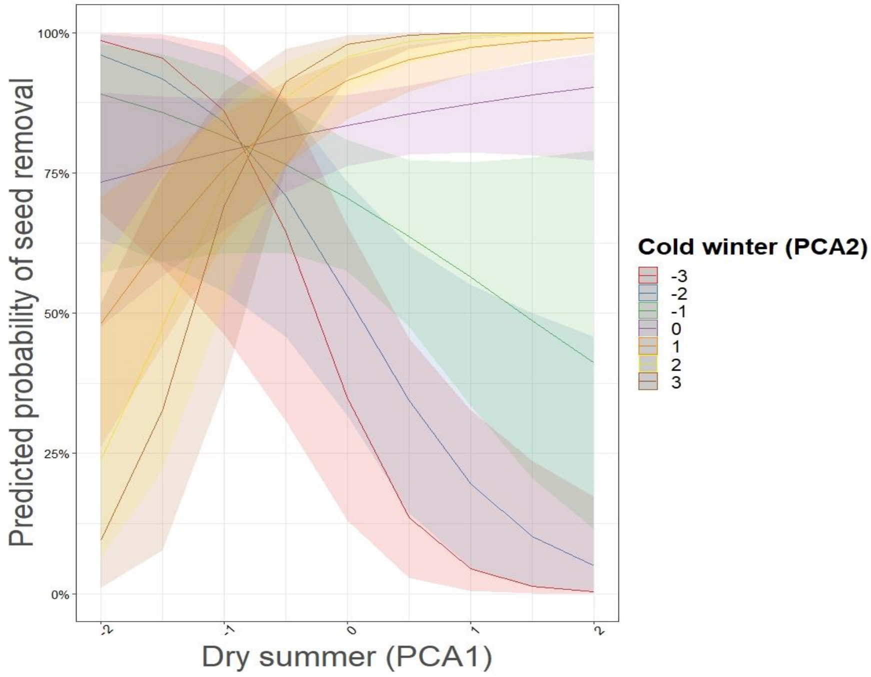

Figure S16 Effects of the interaction between medium-term climatic variables on seed removal in grasslands. Both variables were standardized to mean = 0 and standard deviation = 1.

Table S13 Seed removal in forests

GLMM results (664 observations, 136 plots; 3 regions)				
**Fixed effects***	**Estimate**	**Std.Error**	**t value**	**Pr(>|z|)**
(Intercept)	5.441	0.502	10.844	<0.001***
ST maximum temperature	-0.395	0.393	-1.003	0.316
ST precipitation	-0.158	0.454	-0.348	0.728
**ST temperature: ST precipitation**	**-1.131**	**0.438**	**-2.583**	**0.010****
MT climate: Dry summer (PCA axis1)	-0.242	0.477	-0.508	0.612
**MT climate: Cold winter (PCA axis2)**	**-1.587**	**0.529**	**-3.000**	**0.003****
MT DrySummer: ColdWinter	-0.127	0.490	-0.258	0.796
Wood harvest	0.108	0.464	0.233	0.815
**Non-natural trees**	**-1.312**	**0.436**	**-3.010**	**0.003****
Anthropogenic deadwood	0.712	0.437	1.632	0.103

ST: short-term, MT: medium-term

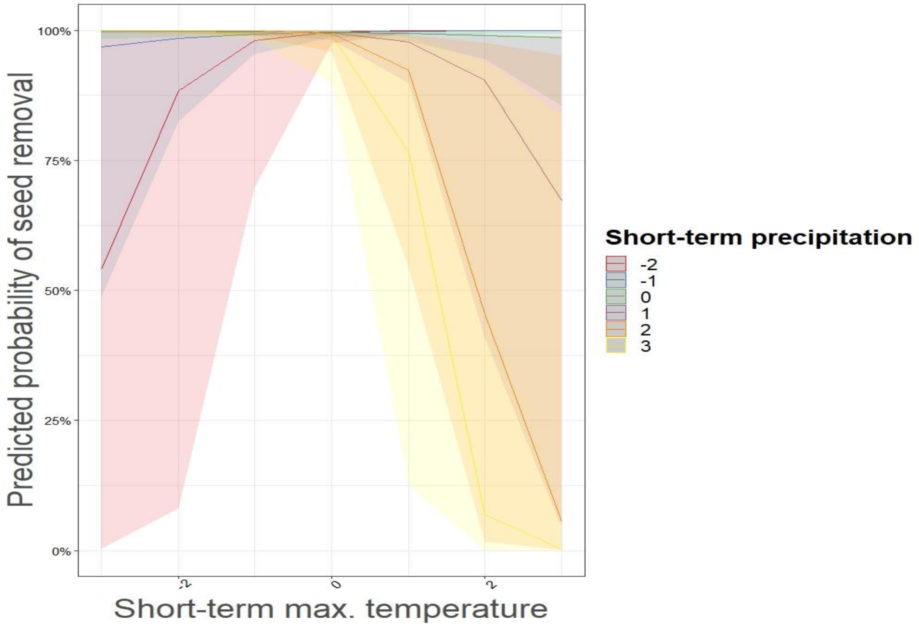

Figure S17 Effects of interaction between the short-term climatic variables on seed removal in forests. Both variables were standardized to mean = 0 and standard deviation = 1.

C. Predation

I. Predation in grasslands

Table S14 Overall predation in grasslands

GLMM results (669 observations, 134 plots, 3 regions)				
**Fixed effects***	**Estimate**	**Std.Error**	**t value**	**Pr(>|z|)**
(Intercept)	-1.309	0.125	-10.488	<0.001***
ST mean temperature	-0.257	0.161	-1.597	0.110
ST precipitation	-0.187	0.151	-1.242	0.214
ST temperature: ST precipitation	0.152	0.274	0.554	0.580
MT climate: Dry summer (PCA axis1)	-0.093	0.137	-0.679	0.497
**MT climate: Cold winter (PCA axis2)**	**0.376**	**0.125**	**3.002**	**0.003*** **
MT DrySummer: ColdWinter	-0.230	0.150	-1.534	0.125
**ST grazing**	**-0.264**	**0.120**	**-2.194**	**0.028***
**ST Mowing**	**-0.322**	**0.127**	**-2.542**	**0.011***
**ST Fertilization**	**0.350**	**0.121**	**2.896**	**0.004****
**MT Grazing**	**-0.246**	**0.116**	**-2.126**	**0.034***

ST: short-term, MT: medium-term

Table S15 Arthropod predation in grasslands

GLMM results (669 observations, 134 plots, 3 regions)				
**Fixed effects**	**Estimate**	**Std.Error**	**t value**	**Pr(>|z|)**
(Intercept)	-1.639	0.135	-12.110	<0.001***
ST mean temperature	-0.318	0.174	-1.825	0.068 .
ST precipitation	-0.199	0.164	-1.211	0.226
ST temperature: ST precipitation	0.172	0.297	0.578	0.563
MT climate: Dry summer (PCA axis1)	-0.102	0.149	-0.686	0.493
**MT climate: Cold winter (PCA axis2)**	**0.363**	**0.135**	**2.683**	**0.007****
MT DrySummer: ColdWinter	-0.263	0.162	-1.622	0.105
ST grazing	-0.104	0.130	-0.798	0.425
ST Mowing	-0.266	0.137	-1.943	0.052 .
**ST Fertilization**	**0.475**	**0.130**	**3.656**	**<0.001*** **
**MT Grazing**	**-0.278**	**0.129**	**-2.154**	**0.031***

ST: short-term, MT: medium-term

Table S16 Bird predation in grasslands

GLMM results (669 observations, 134 plots, 3 regions)				
**Fixed effects**	**Estimate**	**Std.Error**	**t value**	**Pr(>|z|)**
(Intercept)	-9.210	1.251	-7.365	<0.001***
ST mean temperature	-0.288	0.789	-0.365	0.715
ST precipitation	-0.223	0.667	-0.334	0.738
ST temperature: ST precipitation	0.501	1.305	0.384	0.701
MT climate: Dry summer (PCA axis1)	0.565	0.718	0.787	0.431
MT climate: Cold winter (PCA axis2)	1.191	0.632	1.884	0.060 .
MT DrySummer: ColdWinter	-0.628	0.765	-0.820	0.412
ST grazing	-0.924	0.806	-1.146	0.252
ST Mowing	0.483	0.550	0.878	0.380
ST Fertilization	-0.900	0.828	-1.086	0.277

ST: short-term, MT: medium-term

Table S17 Rodent predation in grasslands

GLMM results (669 observations, 134 plots, 3 regions)				
**Fixed effects**	**Estimate**	**Std.Error**	**t value**	**Pr(>|z|)**
(Intercept)	-4.601	0.241	-19.121	<0.001***
ST mean temperature	-0.062	0.240	-0.257	0.797
ST precipitation	-0.049	0.225	-0.220	0.826
ST temperature: ST precipitation	-0.087	0.409	-0.214	0.831
**MT climate: Dry summer (PCA axis1)**	**-0.624**	**0.205**	**-3.045**	**0.002****
MT climate: Cold winter (PCA axis2)	-0.003	0.191	-0.015	0.988
MT DrySummer: ColdWinter	0.322	0.226	1.425	0.154
**ST grazing**	**-0.685**	**0.242**	**-2.825**	**0.005****
**ST Mowing**	**-0.747**	**0.217**	**-3.446**	**0.001*****
ST Fertilization	-0.112	0.194	-0.575	0.566

ST: short-term, MT: medium-term

II. Predation in forests

Table S18 Overall predation in forests

**GLMM results (729 observations, 146 plots; 3 regions)**				
**Fixed effects**	**Estimate**	**Std.Error**	**t value**	**Pr(>|z|)**
(Intercept)	0.087	0.075	1.151	0.250
ST mean temperature	-0.029	0.090	-0.319	0.749
ST precipitation	0.006	0.098	0.066	0.947
ST temperature: ST precipitation	0.057	0.133	0.428	0.669
**MT climate: Dry summer (PCA axis1)**	**-0.272**	**0.088**	**-3.098**	**0.002 ****
MT climate: Cold winter (PCA axis2)	-0.055	0.096	-0.569	0.569
MT DrySummer: ColdWinter	0.004	0.108	0.041	0.967
Wood harvest	0.023	0.090	0.250	0.803
Non-natural trees	-0.013	0.080	-0.165	0.869
Anthropogenic deadwood	0.017	0.083	0.209	0.835

ST: short-term, MT: medium-term

Table S19 Arthropod predation in forests

GLMM results (730 observations, 146 plots; 3 regions)				
**Fixed effects**	**Estimate**	**Std.Error**	**t value**	**Pr(>|z|)**
(Intercept)	-1.089	0.081	-13.490	<0.001***
ST mean temperature	-0.130	0.096	-1.353	0.176
ST precipitation	0.102	0.104	0.984	0.325
**ST temperature: ST precipitation**	**0.287**	**0.141**	**2.037**	**0.042***
MT climate: Dry summer (PCA axis1)	-0.103	0.093	-1.102	0.270
MT climate: Cold winter (PCA axis2)	-0.150	0.104	-1.443	0.149
MT DrySummer: ColdWinter	-0.203	0.117	-1.734	0.083
Wood harvest	-0.132	0.099	-1.338	0.181
**Nonnative tree species**	**0.285**	**0.085**	**3.350**	**0.001***
Anthropogenic deadwood	-0.098	0.089	-1.112	0.266

ST: short-term, MT: medium-term

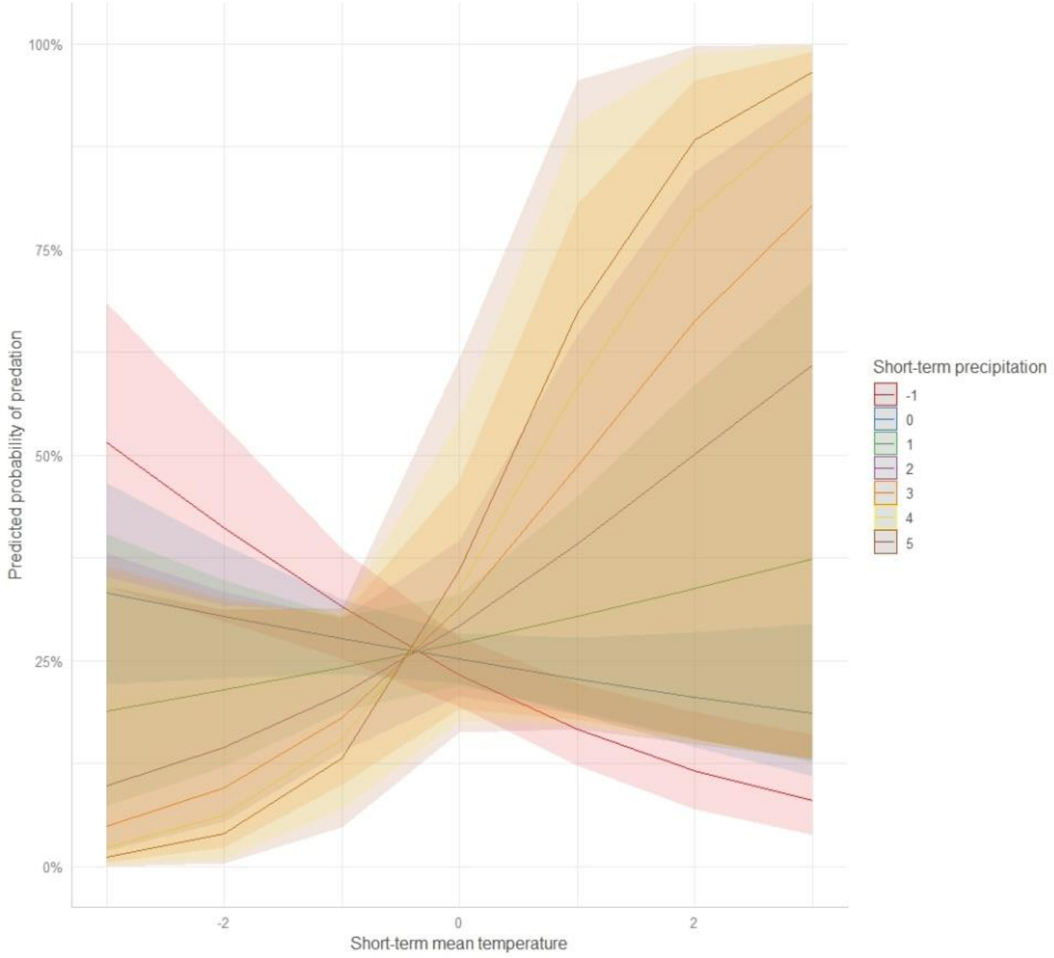

Figure S18 Effects of the interaction between the short-term climatic variables on predation by arthropods in forests. Both variables were standardized to mean = 0 and standard deviation = 1.

Table S20 Bird predation in forests

GLMM results (730 observations, 146 plots, 3 regions)				
**Fixed effects**	**Estimate**	**Std.Error**	**t value**	**Pr(>|z|)**
(Intercept)	-4.401	0.220	-20.008	<0.001***
ST mean temperature	-0.192	0.227	-0.848	0.396
**ST precipitation**	**-0.599**	**0.234**	**-2.561**	**0.010***
ST temperature: ST precipitation	0.174	0.364	0.479	0.632
**MT climate: Dry summer (PCA axis1)**	**1.077**	**0.199**	**5.403**	**<0.001*** **
**MT climate: Cold winter (PCA axis2)**	**0.887**	**0.208**	**4.273**	**<0.001*** **
MT DrySummer: ColdWinter	0.017	0.217	0.079	0.937
**Wood harvest**	**-0.567**	**0.214**	**-2.650**	**0.008****
Non-natural trees	-0.088	0.174	-0.507	0.612
Anthropogenic deadwood	-0.017	0.180	-0.094	0.925

ST: short-term, MT: medium-term

Table S21 Rodent predation in forests

GLMM results (730 observations, 146 plots, 3 regions)				
**Fixed effects**	**Estimate**	**Std.Error**	**t value**	**Pr(>|z|)**
(Intercept)	-1.690	0.446	-3.792	<0.001***
ST mean temperature	0.135	0.127	1.060	0.289
ST precipitation	0.093	0.140	0.665	0.506
ST temperature: ST precipitation	-0.161	0.187	-0.860	0.390
MT climate: Dry summer (PCA axis1)	-0.195	0.321	-0.607	0.544
MT climate: Cold winter (PCA axis2)	0.008	0.224	0.037	0.971
MT DrySummer: ColdWinter	-0.170	0.175	-0.972	0.331
Wood harvest	0.117	0.135	0.867	0.386
**Non-natural trees**	**-0.306**	**0.131**	**-2.326**	**0.020***
**Anthropogenic deadwood**	**0.301**	**0.121**	**2.487**	**0.013***

ST: short-term, MT: medium-term

D. Herbivory

Models were conducted at the plot level. Due to collinearity, short-term precipitation and medium-term fertilization were not included in the models. ST: short-term, MT: medium-term.

Table S22 Total herbivory in grasslands

**GLMM results (145 plots, 3 regions)**				
**Fixed effects**	**Estimate**	**Std.Error**	**t value**	**Pr(>|z|)**
(Intercept)	-4.380	0.243	-18.025	<0.001***
**ST max temperature cumulative**	**-0.223**	**0.066**	**-3.390**	**0.001*****
MT climate: Dry summer (PCA axis1)	0.056	0.198	0.284	0.776
MT climate: Warm winter (PCA axis2)	-0.086	0.059	-1.445	0.148
MT DrySummer:WarmWinter	-0.026	0.052	-0.496	0.620
ST Fertilization	-0.143	0.093	-1.537	0.124
ST Grazing	-0.132	0.076	-1.735	0.083.
MT Grazing	-0.049	0.052	-0.947	0.344
MT Mowing	-0.001	0.064	-0.013	0.990

ST: short-term, MT: medium-term

Table S23 Grass herbivory in grasslands

**GLMM results (143 plots, 3 regions)**				
**Fixed effects**	**Estimate**	**Std.Error**	**t value**	**Pr(>|z|)**
(Intercept)	-5.730	1.073	-5.339	<0.001***
**ST max temperature cumulative**	**-1.260**	**0.278**	**-4.532**	**<0.001*****
MT climate: Dry summer (PCA axis1)	-0.371	0.507	-0.733	0.464
MT climate: Warm winter (PCA axis2)	-0.261	0.171	-1.528	0.127
MT DrySummer:WarmWinter	0.076	0.144	0.528	0.598
ST Fertilization	-0.034	0.108	-0.317	0.751
ST Grazing	-0.255	0.142	-1.796	0.073.
MT Grazing	-0.239	0.165	-1.450	0.147
**MT Mowing**	**-0.367**	**0.108**	**-3.414**	**0.001*****

ST: short-term, MT: medium-term

Table S24 Herb herbivory in grasslands

**GLMM results (140 plots, 3 regions)**				
**Fixed effects**	**Estimate**	**Std.Error**	**t value**	**Pr(>|z|)**
(Intercept)	-4.222	0.234	-18.025	<0.001***
ST max temperature cumulative	**-0.253**	**0.079**	**-3.191**	**0.001****
MT climate: Dry summer (PCA axis1)	0.398	0.220	1.808	0.071.
MT climate: Warm winter (PCA axis2)	-0.052	0.094	-0.549	0.583
MT DrySummer:WarmWinter	0.048	0.080	0.605	0.545
ST Fertilization	-0.164	0.130	-1.265	0.206
**ST Grazing**	**-0.304**	**0.152**	**-1.997**	**0.046***
MT Grazing	0.014	0.058	0.233	0.815
MT Mowing	0.119	0.078	1.518	0.129

ST: short-term, MT: medium-term

Table S25 Total herbivory in forests

**GLMM results (146 plots, 3 regions)**				
**Fixed effects**	**Estimate**	**Std.Error**	**t value**	**Pr(>|z|)**
(Intercept)	-3.273	0.048	-67.680	<0.001***
**ST max temperature cumulative**	**-0.139**	**0.054**	**-2.548**	**0.011***
**MT climate: Dry summer (PCA axis1)**	**0.174**	**0.042**	**4.116**	**<0.001*****
MT climate: Warm winter (PCA axis2)	0.034	0.046	0.736	0.462
MT Dry Summer:Warm Winter	-0.017	0.038	-0.443	0.658
**Wood harvest**	**-0.100**	**0.047**	**-2.113**	**0.035***
**Non-natural trees**	**-0.460**	**0.068**	**-6.789**	**<0.001*****
Anthropogenic deadwood	-0.013	0.036	-0.356	0.722

ST: short-term, MT: medium-term

SUPPLEMENTARY MATERIAL S5 Comparison of the mean effect sizes of short-term vs. medium-term land-use variables in grasslands

This section aims to compare the effect sizes of land-use variables in the short-term vs. medium-term timescales. As those variables were correlated within and among each variable set, we did not analyze them together but we obtained the mean effect sizes from separate models using either short-term or medium-term variables together with the climatic conditions, following the main model structure indicated in the Methods. As we focused on the short-term effects in the main findings, we used the results presented in the Results and the Supplementary material S6 for the short-term effects of dung removal, seed removal, predation by birds or rodents. In case that those results included the medium-term effects such as the case for some predation and herbivory tests, we also run a model only with the short-term effects and then only with the medium-term effects of the corresponding variables. The medium-term variables included grazing and fertilization intensities but not of mowing due to collinearity. The short-term variables included all three land-use components except for herbivory as the subplots that herbivory measurements took place were not mown. We then calculated the mean effect size of land-use variable sets, the absolute of each effect size weighted inversely by its standard error. The results show that the mean effect sizes of short-term land-use variables were higher than that of the medium-term variables for all processes except for the seed removal and the predation by the arthropods (Fig. S19).

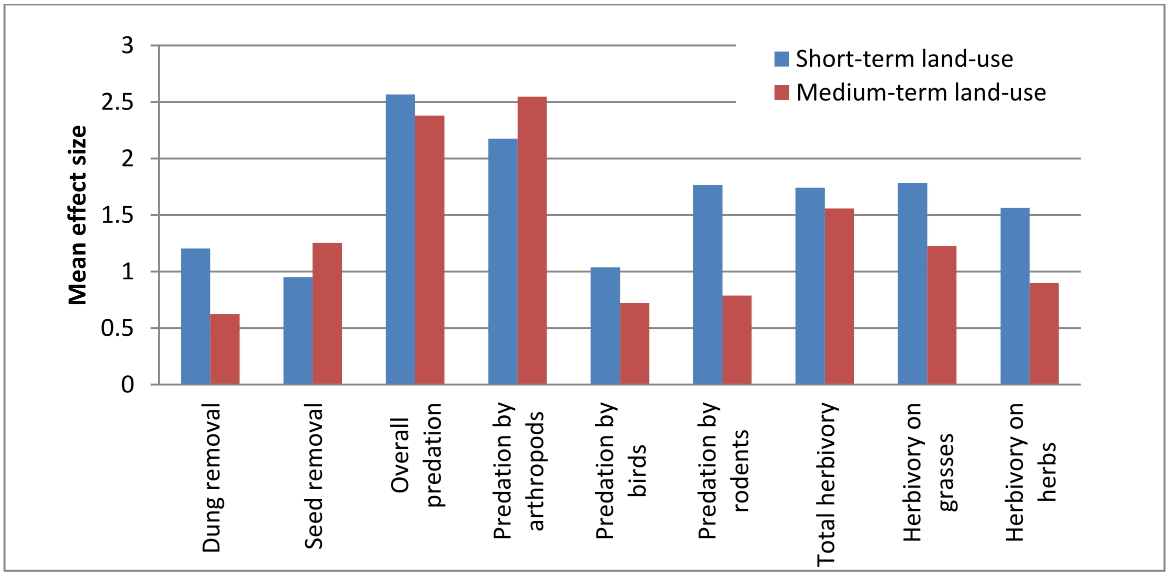

Figure S19 Mean effect sizes of short-term (blue bars) vs. medium-term (red bars) land-use components in grasslands. The effect sizes were obtained from the GLMM models and weighted inversely by their standard errors.

Results of the additional GLMMs used to calculate the mean effect sizes of the land-use variables on the process rates in grasslands

Table S26 Dung removal analysis with the climatic and medium-term land-use variables

**GLMM results (657 observations, 134 plots, 3 regions)**					
**Fixed effects***	**Estimate**	**Std.Error**	**t value**	**Pr(>|z|)**	
(Intercept)	0.132	0.015	8.697	<0.001	***
ST maximum temperature	-0.011	0.019	-0.590	0.555	
**ST precipitation**	**0.037**	**0.017**	**2.103**	**0.035**	*
ST temperature: ST precipitation	-0.019	0.035	-0.543	0.587	
**MT climate: Dry summer (PCA axis1)**	**0.031**	**0.014**	**2.174**	**0.030**	*
MT climate: Cold winter (PCA axis2)	-0.019	0.013	-1.469	0.142	
MT DrySummer: ColdWinter	-0.018	0.016	-1.109	0.268	
MT Grazing	0.006	0.012	0.479	0.632	
MT Fertilization	-0.010	0.013	-0.768	0.442	

ST: short-term, MT: medium-term

Table S27 Seed removal analysis with the climatic and medium-term land-use variables

**GLMM results (657 observations, 134 plots, 3 regions)**					
**Fixed effects***	**Estimate**	**Std.Error**	**t value**	**Pr(>|z|)**	
(Intercept)	1.586	0.231	6.869	<0.001	***
ST maximum temperature	-0.078	0.228	-0.341	0.733	
ST precipitation	0.397	0.224	1.771	0.076	.
ST temperature: ST precipitation	0.414	0.333	1.243	0.214	
MT climate: Dry summer (PCA axis1)	0.345	0.253	1.364	0.173	
**MT climate: Cold winter (PCA axis2)**	**0.796**	**0.213**	**3.738**	**<0.001**	***
**MT DrySummer: ColdWinter**	**0.874**	**0.259**	**3.380**	**0.001**	***
MT Grazing	-0.314	0.182	-1.723	0.085	.
MT Fertilization	-0.147	0.186	-0.790	0.429	

ST: short-term, MT: medium-term

Table S28 Overall predation analysis with the climatic and medium-term land-use variables

**GLMM results (669 observations, 134 plots, 3 regions)**					
**Fixed effects***	**Estimate**	**Std.Error**	**t value**	**Pr(>|z|)**	
(Intercept)	-1.285	0.130	-9.924	<0.001	***
**ST mean temperature**	**-0.330**	**0.164**	**-2.017**	**0.044**	*
ST precipitation	-0.285	0.153	-1.871	0.061	.
ST temperature: ST precipitation	0.285	0.290	0.981	0.326	
MT climate: Dry summer (PCA axis1)	-0.151	0.140	-1.076	0.282	
**MT climate: Cold winter (PCA axis2)**	**0.390**	**0.127**	**3.085**	**0.002**	**
MT DrySummer: ColdWinter	-0.240	0.155	-1.552	0.121	
**MT Grazing**	**-0.236**	**0.116**	**-2.039**	**0.041**	*
**MT Fertilization**	**0.328**	**0.120**	**2.721**	**0.007**	**

ST: short-term, MT: medium-term

Table S29 Overall predation analysis with the climatic and only short-term land-use variables

GLMM results (669 observations, 134 plots, 3 regions)					
**Fixed effects***	**Estimate**	**Std.Error**	**t value**	**Pr(>|z|)**	
(Intercept)	-1.309	0.127	-10.312	<0.001	***
ST maximum temperature	-0.273	0.163	-1.670	0.095	.
ST precipitation	-0.181	0.153	-1.185	0.236	
ST temperature: ST precipitation	0.120	0.278	0.433	0.665	
MT climate: Dry summer (PCA axis1)	-0.147	0.137	-1.076	0.282	
**MT climate: Cold winter (PCA axis2)**	**0.358**	**0.127**	**2.815**	**0.005**	**
MT DrySummer: ColdWinter	-0.206	0.152	-1.349	0.177	
**ST Grazing**	**-0.315**	**0.120**	**-2.625**	**0.009**	**
**ST Mowing**	**-0.288**	**0.128**	**-2.259**	**0.024**	*

ST: short-term, MT: medium-term

Table S30 Arthropod predation analysis with the climatic and medium-term land-use variables

GLMM results (669 observations, 134 plots, 3 regions)					
**Fixed effects***	**Estimate**	**Std.Error**	**t value**	**Pr(>|z|)**	
(Intercept)	-1.620	0.138	-11.709	<0.001	***
**ST mean temperature**	**-0.389**	**0.174**	**-2.228**	**0.026**	*
ST precipitation	-0.290	0.164	-1.776	0.076	.
ST temperature: ST precipitation	0.283	0.310	0.911	0.362	
MT climate: Dry summer (PCA axis1)	-0.164	0.149	-1.099	0.272	
**MT climate: Cold winter (PCA axis2)**	**0.339**	**0.134**	**2.528**	**0.011**	*
MT DrySummer: ColdWinter	-0.283	0.165	-1.719	0.086	.
MT Grazing	-0.240	0.126	-1.899	0.058	.
**MT Fertilization**	**0.407**	**0.127**	**3.197**	**0.001**	**

ST: short-term, MT: medium-term

Table S31 Arthropod predation analysis with the climatic and short-term land-use variables

GLMM results (669 observations, 134 plots, 3 regions)					
**Fixed effects***	**Estimate**	**Std.Error**	**t value**	**Pr(>|z|)**	
(Intercept)	-1.620	0.138	-11.709	<0.001	***
**ST mean temperature**	**-0.389**	**0.174**	**-2.228**	**0.026**	*
ST precipitation	-0.290	0.164	-1.776	0.076	.
ST temperature: ST precipitation	0.283	0.310	0.911	0.362	
MT climate: Dry summer (PCA axis1)	-0.164	0.149	-1.099	0.272	
**MT climate: Cold winter (PCA axis2)**	**0.339**	**0.134**	**2.528**	**0.011**	*
MT DrySummer: ColdWinter	-0.283	0.165	-1.719	0.086	.
MT Grazing	-0.240	0.126	-1.899	0.058	.
**MT Fertilization**	**0.407**	**0.127**	**3.197**	**0.001**	**

ST: short-term, MT: medium-term

Table S32 Bird predation analysis with the climatic and medium-term land-use variables

GLMM results (669 observations, 134 plots, 3 regions)					
**Fixed effects***	**Estimate**	**Std.Error**	**t value**	**Pr(>|z|)**	
(Intercept)	-9.656	1.261	-7.655	<0.001	***
ST maximum temperature	-0.148	0.790	-0.187	0.852	
ST precipitation	-0.065	0.649	-0.101	0.920	
ST temperature: ST precipitation	0.532	1.400	0.380	0.704	
MT climate: Dry summer (PCA axis1)	0.558	0.753	0.741	0.459	
MT climate: Cold winter (PCA axis2)	1.086	0.635	1.711	0.087	.
MT DrySummer: ColdWinter	-0.640	0.822	-0.779	0.436	
MT Grazing	-0.237	0.503	-0.470	0.638	
MT Fertilization	-1.479	1.511	-0.979	0.328	

ST: short-term, MT: medium-term

Table S33 Rodent predation analysis with the climatic and medium-term land-use variables

GLMM results (669 observations, 134 plots, 3 regions)					
**Fixed effects***	**Estimate**	**Std.Error**	**t value**	**Pr(>|z|)**	
(Intercept)	-4.612	0.259	-17.790	<0.001	***
ST maximum temperature	-0.152	0.268	-0.567	0.571	
ST precipitation	-0.225	0.246	-0.914	0.361	
ST temperature: ST precipitation	0.011	0.464	0.023	0.982	
**MT climate: Dry summer (PCA axis1)**	**-0.626**	**0.225**	**-2.788**	**0.005**	**
MT climate: Cold winter (PCA axis2)	0.096	0.205	0.467	0.640	
MT DrySummer: ColdWinter	0.271	0.248	1.096	0.273	
MT Grazing	-0.211	0.191	-1.104	0.270	
MT Fertilization	-0.094	0.199	-0.471	0.637	

ST: short-term, MT: medium-term

Table S34 Total herbivory analysis with the climatic and medium-term land-use variables

GLMM results (145 plots, 3 regions)					
**Fixed effects***	**Estimate**	**Std.Error**	**t value**	**Pr(>|z|)**	
(Intercept)	-4.364	0.223	-19.531	<0.001	***
**ST max temperature cumulative**	**-0.223**	**0.063**	**-3.513**	**<0.001**	***
MT climate: Dry summer (PCA axis1)	0.051	0.208	0.245	0.806	
MT climate: Warm winter (PCA axis2)	-0.063	0.059	-1.057	0.290	
MT DrySummer:WarmWinter	-0.026	0.052	-0.498	0.618	
MT Grazing	-0.070	0.051	-1.368	0.171	
MT Fertilization	-0.195	0.111	-1.749	0.080	.

ST: short-term, MT: medium-term

Table S35 Total herbivory analysis with the climatic and short-term land-use variables

GLMM results (145 plots, 3 regions)					
**Fixed effects***	**Estimate**	**Std.Error**	**t value**	**Pr(>|z|)**	
(Intercept)	-4.376	0.246	-17.760	<0.001	***
**ST max temperature cumulative**	**-0.215**	**0.065**	**-3.286**	**0.001**	**
MT climate: Dry summer (PCA axis1)	0.002	0.205	0.008	0.994	
MT climate: Warm winter (PCA axis2)	-0.070	0.057	-1.223	0.221	
MT DrySummer:WarmWinter	-0.009	0.050	-0.174	0.862	
ST Fertilization	-0.144	0.088	-1.631	0.103	
ST Grazing	-0.140	0.076	-1.855	0.064	.

ST: short-term, MT: medium-term

Table S36 Grass herbivory analysis with the climatic and medium-term land-use variables

GLMM results (143 plots, 3 regions)					
**Fixed effects***	**Estimate**	**Std.Error**	**t value**	**Pr(>|z|)**	
(Intercept)	-5.424	1.186	-4.574	<0.001	***
**ST max temperature cumulative**	**-0.818**	**0.401**	**-2.041**	**0.041**	*
MT climate: Dry summer (PCA axis1)	-0.684	0.609	-1.123	0.261	
MT climate: Warm winter (PCA axis2)	-0.160	0.158	-1.015	0.310	
MT DrySummer:WarmWinter	0.131	0.167	0.787	0.431	
MT Grazing	-0.212	0.185	-1.147	0.251	
MT Fertilization	-1.500	1.151	-1.303	0.193	

ST: short-term, MT: medium-term

Table S37 Grass herbivory analysis with the climatic and short-term land-use variables

GLMM results (143 plots, 3 regions)					
**Fixed effects***	**Estimate**	**Std.Error**	**t value**	**Pr(>|z|)**	
(Intercept)	-5.308	1.230	-4.316	0.000	***
**ST max temperature cumulative**	**-1.124**	**0.299**	**-3.755**	**0.000**	***
MT climate: Dry summer (PCA axis1)	-1.112	0.570	-1.951	0.051	.
MT climate: Warm winter (PCA axis2)	-0.268	0.158	-1.691	0.091	.
MT DrySummer:WarmWinter	0.270	0.154	1.752	0.080	.
**ST Fertilization**	**-0.200**	**0.100**	**-1.997**	**0.046**	*
ST Grazing	-0.199	0.127	-1.568	0.117	

ST: short-term, MT: medium-term

Table S38 Herb herbivory analysis with the climatic and medium-term land-use variables

GLMM results (140 plots, 3 regions)					
**Fixed effects***	**Estimate**	**Std.Error**	**t value**	**Pr(>|z|)**	
(Intercept)	-4.148	0.174	-23.832	<0.001	***
**ST max temperature cumulative**	**-0.242**	**0.076**	**-3.173**	**0.002**	**
MT climate: Dry summer (PCA axis1)	0.303	0.164	1.849	0.064	.
MT climate: Warm winter (PCA axis2)	0.024	0.097	0.251	0.802	
MT DrySummer:WarmWinter	0.089	0.085	1.050	0.294	
MT Grazing	-0.046	0.065	-0.707	0.479	
MT Fertilization	-0.186	0.171	-1.090	0.276	

ST: short-term, MT: medium-term

Table S39 Herb herbivory analysis with climatic and short-term land-use variables

GLMM results (140 plots, 3 regions)					
**Fixed effects***	**Estimate**	**Std.Error**	**t value**	**Pr(>|z|)**	
(Intercept)	-4.202	0.223	-18.866	< 2e-16	***
**ST max temperature cumulative**	**-0.243**	**0.078**	**-3.119**	**0.002**	**
MT climate: Dry summer (PCA axis1)	0.349	0.195	1.789	0.074	.
MT climate: Warm winter (PCA axis2)	-0.024	0.089	-0.267	0.789	
MT DrySummer:WarmWinter	0.054	0.079	0.690	0.490	
ST Fertilization	-0.115	0.118	-0.972	0.331	
**ST Grazing**	**-0.324**	**0.150**	**-2.157**	**0.031**	*

ST: short-term, MT: medium-term

SUPPLEMENTARY MATERIAL S6 Process rates in detail

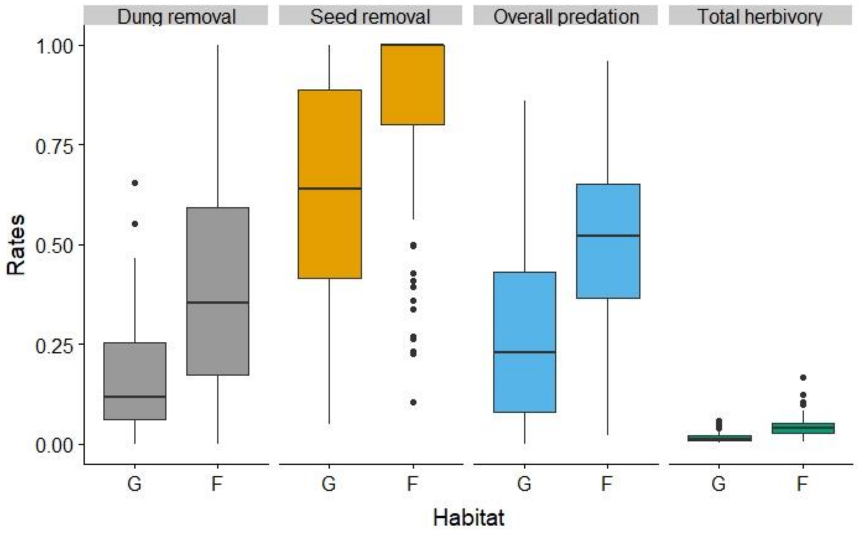

Figure S20 Boxplots showing plot-level mean rates of major processes in forests and grasslands. G: Grassland, F: Forest.

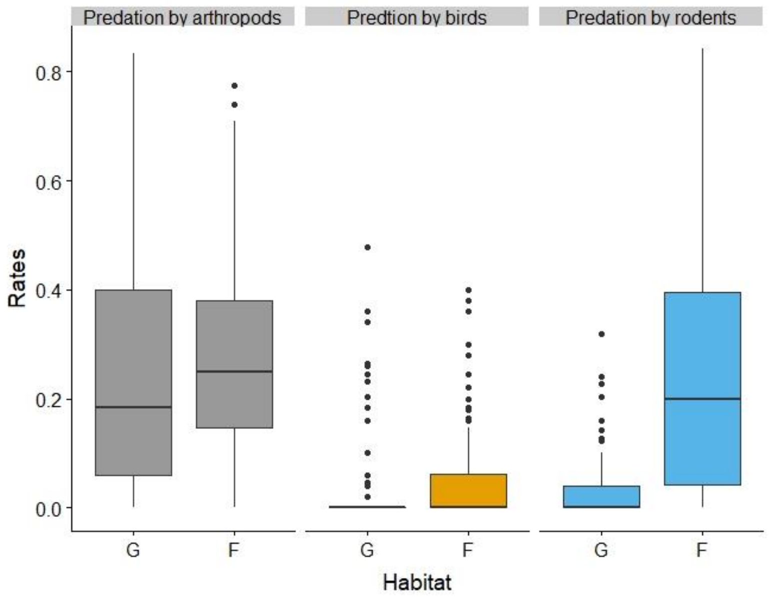

Figure S21 Boxplots of plot-level predation rates by each predator group in two habitats. Predation by each group was calculated as the proportion of caterpillars with at least one predation mark by each group. G: Grassland, F: Forest.

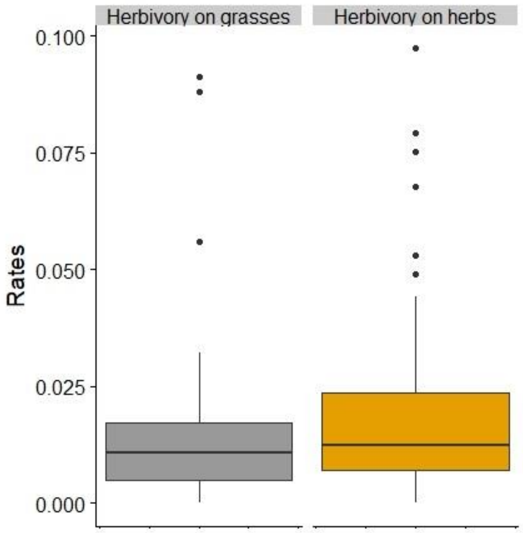

Figure S22 Boxplots of plot-level herbivory rates on two plant groups in grasslands.

Table S40 Results of ANOVA tests on the plot-level process rates between forests and grasslands. Df: degrees of freedom, Sum Sq: sum of squares, Mean Sq: Mean squares. Herbivory on grasses and on herbs are not included as they were measured only in grasslands.

**Processes**		**Df**	**Sum Sq**	**Mean Sq**	**F value**	**Pr(>F)**
**Dung removal**	Habitat	1	3.956	3.956	79.57	<2e-16
	Residuals	281	13.972	0.05		
**Seed removal**	Habitat	1	3.646	3.646	61.35	1.22e-13
	Residuals	260	15.453	0.059		
**Predation - overall**	Habitat	1	3.72	3.720	81.2	<2e-16
	Residuals	281	12.87	0.046		
**Predation by arthropods**	Habitat	1	0.081	0.081	2.002	0.158
	Residuals	281	11.389	0.041		
**Predation by birds**	Habitat	1	0.0358	0.036	5.526	0.0194
	Residuals	281	1.819	0.007		
**Predation by rodents**	Habitat	1	3.44	3.44	125	<2e-16
	Residuals	281	7.734	0.028		
**Herbivory**	Habitat	1	0.05377	0.054	156.5	<2e-16
	Residuals	293	0.10071	0.00034		

SUPPLEMENTARY MATERIAL S7: Effects of vegetation structure on process rates

We recorded vegetation structure parameters at the subplot level: vegetation height was measured for the majority (90%) of the ground cover in cm. Vegetation cover of ground layer and canopy density were recorded as percentages. Tables S41 & 42 show the results of GLMM analyses for the effect of vegetation on the process rates at the subplot level. We used all vegetation parameters and region (representing environmental differences between the regions) as fixed effects. We included plot as a random effect (1 | Plot). All numerical explanatory variables were standardized. Significance (Sig.) codes: (“.” : p≤0.1, *: p≤0.05,**: p≤0.01,***: p≤ 0.001. No vegetation structure data are available from herbivory surveys.

Table S41 Results of GLMM analyses for the effect of vegetation on process rates at subplot level in grasslands

Processes:	Dung removal			Seed removal			Predation-overall			Predation by arthropods			Predation by birds			Predation by rodents		
	Estimate	p-value	Sig.	Estimate	p-value	Sig.	Estimate	p-value	Sig.	Estimate	p-value	Sig.	Estimate	p-value	Sig.	Estimate	p-value	Sig.
Intercept	0.111	<0.001	***	0.449	0.159		-0.773	<0.001	***	-1.108	<0.001	***	-8.595	<0.001	***	-3.952	<0.001	***
Vegetation height	-0.012	0.192		0.269	<0.001	***	0.201	0.005	**	0.160	0.033	*	-0.120	0.510		0.637	<0.001	***
Vegetation cover	0.009	0.318		-0.041	0.382		0.135	0.059	.	0.131	0.084	.	0.660	0.044	*	0.039	0.798	
Region Hainich	0.038	0.198		0.434	0.314		-0.649	0.023	*	-0.502	0.100		-2.579	0.095	.	-0.667	0.071	.
Region Schorfheide	0.062	0.065	.	2.405	<0.001	***	-1.070	0.001	***	-1.246	<0.001	***	0.324	0.779		-1.567	<0.001	***

Table S42 Results of GLMM analyses for the effect of vegetation on process rates at subplot level in forests

Processes:	Dung removal			Seed removal			Predation-overall			Predation by arthropods			Predation by birds			Predation by rodents		
	Estimate	p-value	Sig.	Estimate	p-value	Sig.	Estimate	p-value	Sig.	Estimate	p-value	Sig.	Estimate	p-value	Sig.	Estimate	p-value	Sig.
Intercept	0.274	<0.001	***	4.737	<0.001	***	0.298	0.011	*	-1.113	<0.001	***	-4.908	<0.001	***	-0.783	<0.001	***
Vegetation height	-0.002	0.834		-0.254	<0.001	***	0.075	0.022	*	0.002	0.968		-0.072	0.499		0.089	0.009	**
Vegetation cover	0.015	0.184		0.538	<0.001	***	0.100	0.054	.	0.093	0.114		0.137	0.247		0.040	0.543	
Canopy density	-0.016	0.054	.	-0.033	0.549		0.094	0.021	*	0.006	0.904		0.178	0.064	.	0.136	0.009	**
Region Hainich	-0.081	0.042	*	4.484	<0.001	***	-0.114	0.500		-0.123	0.550		-0.865	0.054	.	-0.185	0.451	
Region Shorfheide	0.185	<0.001	***	-1.625	0.090	.	-0.620	0.000	***	0.074	0.717		2.445	<0.001	***	-2.433	<0.001	***

SUPPLEMENTARY MATERIAL S8 Linear correlations between the process rates

Table S43 Linear correlations of the plot-level process rates in grasslands based on Pearson's correlation. *r*: correlation coefficient, *p*: significance level. Correlations between predation by different groups and between different herbivory measures are shown in rectangles for the ease of interpretation.

**Processes in grasslands**	Dung removal		Seed removal		Predation - overall		Predation by arthropods		Predation by birds		Predation by rodents		Herbivory - total		Herbivory on grasses	
	r	*p*	*r*	*p*	*r*	*p*	*r*	*p*	*r*	*p*	*r*	*p*	*r*	*p*	*r*	*p*
Dung removal																
Seed removal	0.040	0.688														
Predation - overall	-0.080	0.371	0.100	0.274												
Predation by arthropods	-0.080	0.386	0.050	0.591	**0.950**	**<0.001**										
Predation by birds	0.030	0.733	0.060	0.529	**0.330**	**<0.001**	0.080	0.362								
Predation by rodents	-0.150	0.079	0.140	0.129	**0.320**	**<0.001**	0.120	0.346	**0.170**	**0.048**						
Herbivory - total	-0.010	0.942	0.080	0.371	-0.170	0.057	-0.150	0.081	0.000	0.986	**-0.190**	**0.036**				
Herbivory on grasses	-0.010	0.880	-0.110	0.215	**-0.190**	**0.033**	-0.150	0.087	-0.130	0.133	-0.080	0.346	**0.510**	**<0.001**		
Herbivory on herbs	-0.050	0.613	**0.200**	**0.027**	-0.070	0.408	-0.110	0.230	0.170	0.062	-0.120	0.175	**0.740**	**<0.001**	0.110	0.204

Table S44 Linear correlations of the plot-level process rates in forests based on Pearson's correlation. *r*: correlation coefficient, *p*: significance level. Correlations between predation by different groups are shown in rectangles for the ease of interpretation.

**Processes in forests**	Dung removal		Seed removal		Predation - overall		Predation by arthropods		Predation by birds		Predation by rodents	
	r	*p*	*r*	*p*	*r*	*p*	*r*	*p*	*r*	*p*	*r*	*p*
Dung removal												
Seed removal	**-0.260**	**0.002**										
Predation - overall	-0.060	0.434	**0.320**	**<0.001**								
Predation by arthropods	**0.180**	**0.031**	-0.020	0.820	**0.480**	**<0.001**						
Predation by birds	**0.410**	**<0.001**	**-0.280**	**0.001**	0.090	0.301	**0.170**	**0.043**				
Predation by rodents	**-0.320**	**<0.001**	**0.440**	**<0.001**	**0.600**	**<0.001**	**-0.330**	**<0.001**	**-0.350**	**<0.001**		
Herbivory - total	-0.110	0.182	0.090	0.280	-0.030	0.745	-0.040	0.630	0.010	0.866	0.000	0.967

SUPPLEMENTARY MATERIAL S9: Within-plot variation of the process rates

We used the coefficient of variation (CV) as a measure of variation in the process rates. We focused on the within-plot variation of each process in each habitat and calculated as CV_{within} = mean (CVplot) = mean (SDplot/ $\bar{x}$plot*100) where SD is the standard deviation and $\bar{x}$ is the mean process rate. We used CV as it is independent of the magnitude of the mean and does not require any assumption on data distribution. Although each process rate was calculated as proportion and varied between 0 and 1 (0-100 in percentage), the histograms of the rates below (Figures S23- S29) did not follow a clear binomial distribution because it is not the random occurrence of a probability but the rate of a natural process affected by various drivers. Our data cover a wide range of means and CVs and the CV decreases as the mean increases, as expected based on the above formula. So there is no drawback in reporting CVs and checking their correlations with land-use intensities as our sample sizes are very large.

Table S45 Percent coefficient of variation of each process rate (CV_{within}) within plots in grasslands and forests. Dung removal, seed removal and predation measurements were based on 5 subplots per plot; herbivory was based on 2 subplots in grasslands, and there were no subplot-level measurements for herbivory in forests. For herbivory in grasslands, mowing did not take place in the short-term timescale, hence LUI includes only grazing and fertilization.

**Processes**	**Habitat**	**CV_{within}**
Dung removal	Forest	69 ± 0.55
Seed removal	Forest	19.5±0.46
Predation – overall	Forest	45.8±0.44
Predation by arthropods	Forest	84±0.56
Predation by birds	Forest	130.9±1.01
Predation by rodents	Forest	106.9±0.7
Dung removal	Grassland	89.7±0.62
Seed removal	Grassland	43.9±0.6
Predation – overall	Grassland	88.46±0.69
Predation by arthropods	Grassland	94.3±0.7
Predation by birds	Grassland	143±1.82
Predation by rodents	Grassland	177.5±1
Herbivory – total	Grassland	37.2±0.44
Herbivory – grasses	Grassland	47.9±0.51
Herbivory – herbs	Grassland	56.1±0.52

Table S46 Correlations between within-plot level variations of process rates (CV_{within} in %) and land-use intensities. Correlation coefficients of Pearson correlation tests were given and significant results are indicated in bold with p values in parentheses. For land-use intensity in forests, a compound measure, ForMI (Kahl and Bauhus, 2014), was used which combines harvest intensity, proportion of non-natural trees in the tree composition, and anthropogenic deadwood. For grasslands, a compound measure, LUI (Blüthgen and others, 2012), combining the intensity of livestock grazing, the number of cuts and fertilization was adopted and the dataset was produced based on the short- and medium-terms of the study. Herbivory in forests was measured at the plot level so no CV_{within} was available.

CV_{within}	Correlation with ForMI	Correlation with short-term LUI	Correlation with medium-term LUI
Dung removal	-0.072	**-0.176 (*p* = 0.04)**	0.095
Seed removal	0.147	**0.340 (*p* < 0.001)**	**0.236 (*p*= 0.008)**
Predation –overall	0.061	0.159	-0.135
Predation by arthropods	0.053	0.124	**-0.211 (*p* = 0.019)**
Predation by birds	-0.174	0.184	0.262
Predation by rodents	-0.038	0.250 (*p*= 0.058)	0.031
Herbivory –overall	NA	0.062	0.117
Herbivory on grasses	NA	0.139	**0.297 (*p* = 0.002)**
Herbivory on herbs	NA	0.150	-0.053

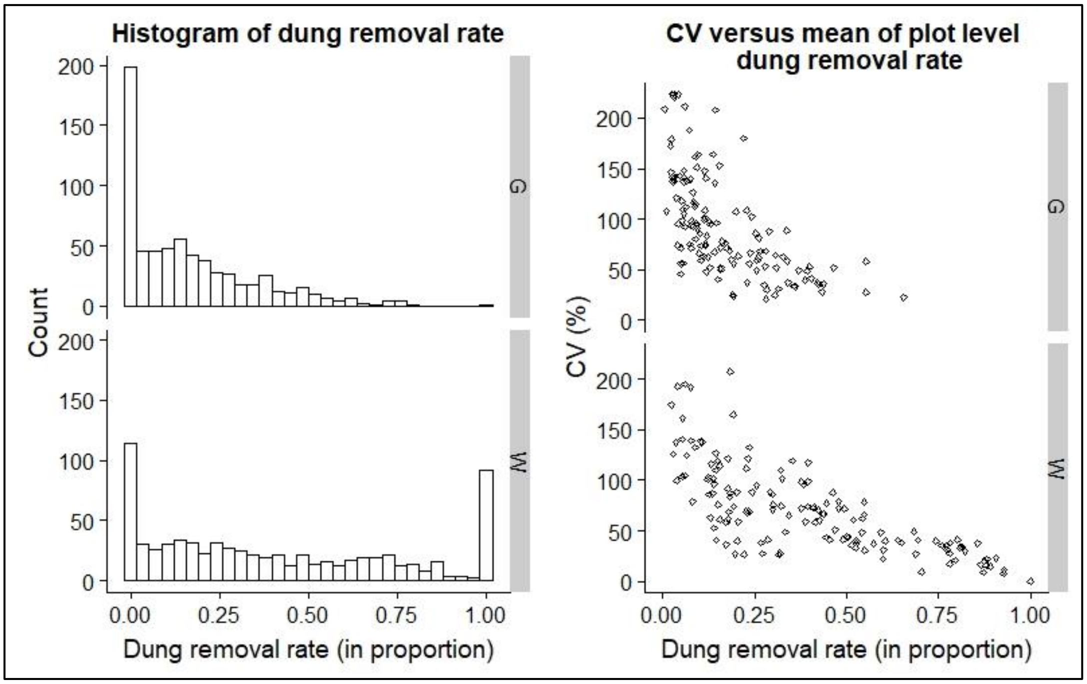

Figure S23 Left: histogram showing subplot level dung removal rates; right: scatterplot showing plot level mean rates and CV for grasslands (G) and forests (W).

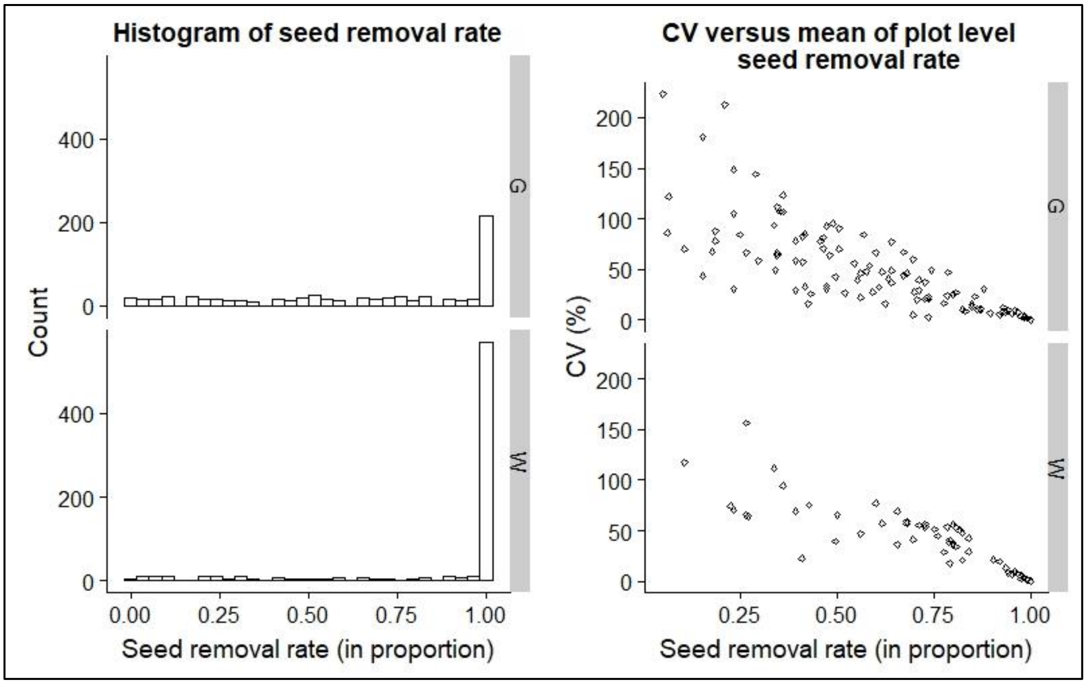

Figure S24 Left: histogram showing subplot level seed removal rates; right: scatterplot showing plot level mean rates and CV for grasslands (G) and forests (W).

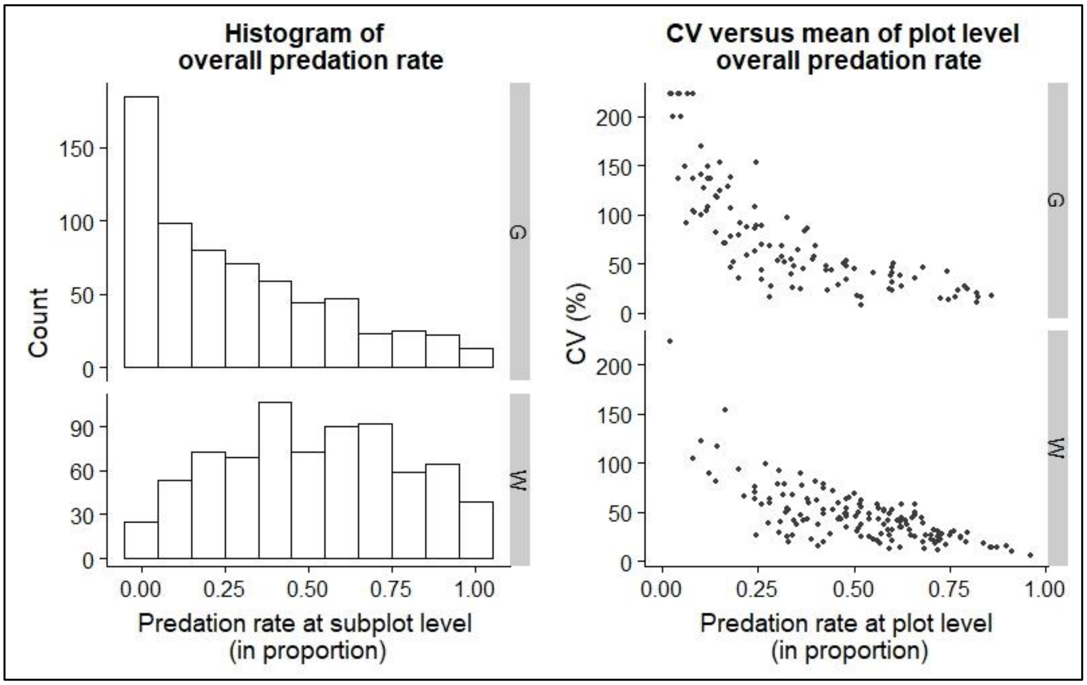

Figure S25 Left: histogram showing subplot level overall predation rates; right: scatterplot showing plot level mean rates and CV for grasslands (G) and forests (W).

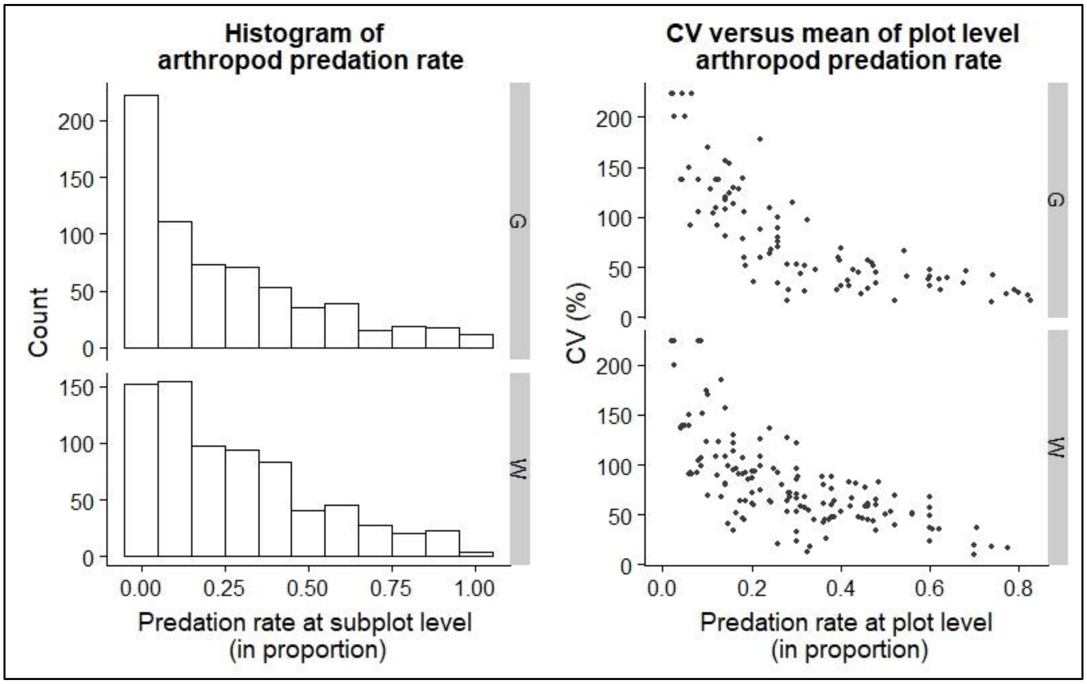

Figure S26 Left: histogram showing subplot level rates of predation by arthropods; right: scatterplot showing plot level mean rates and CV for grasslands (G) and forests (W).

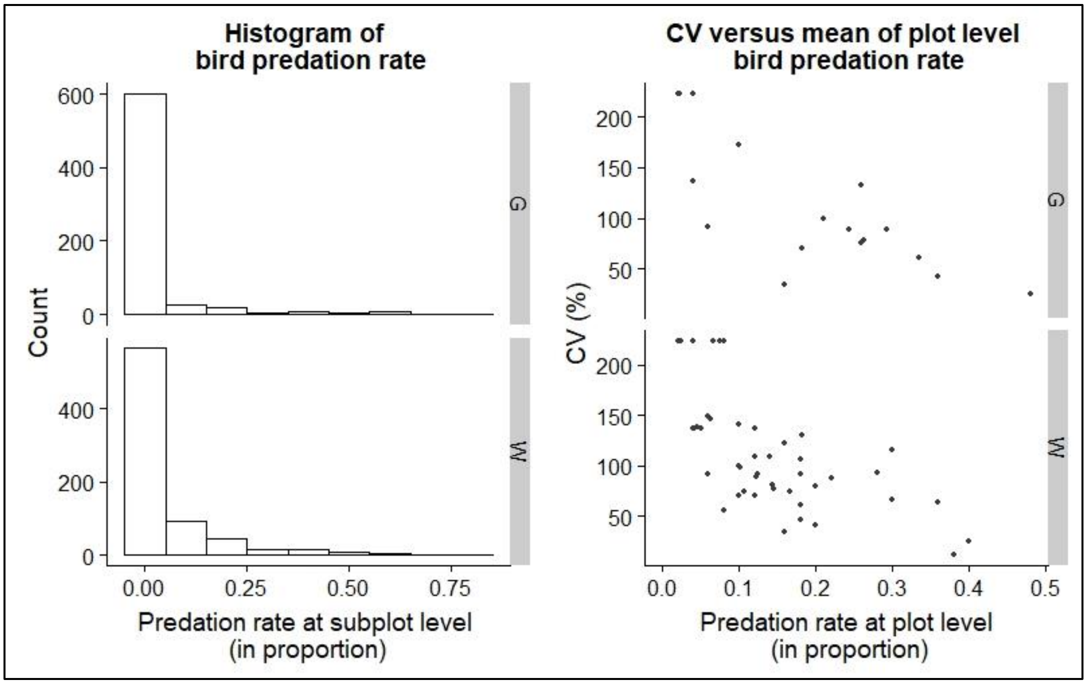

Figure S27 On the left: histogram showing subplot level rates of predation by birds; on the right: scatterplot showing plot level mean rates and CV for grasslands (G) and forests (W).

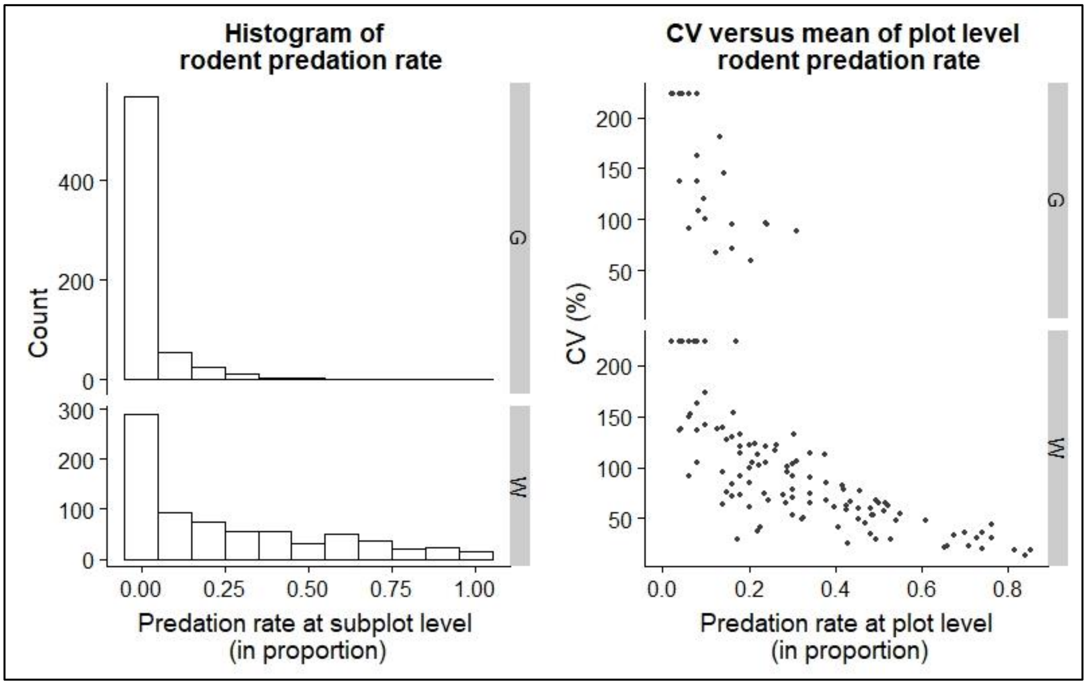

Figure S28 On the left: histogram showing subplot level rates of rodent predation; on the right: scatterplot showing plot level mean rates and CV for grasslands (G) and forests (W).

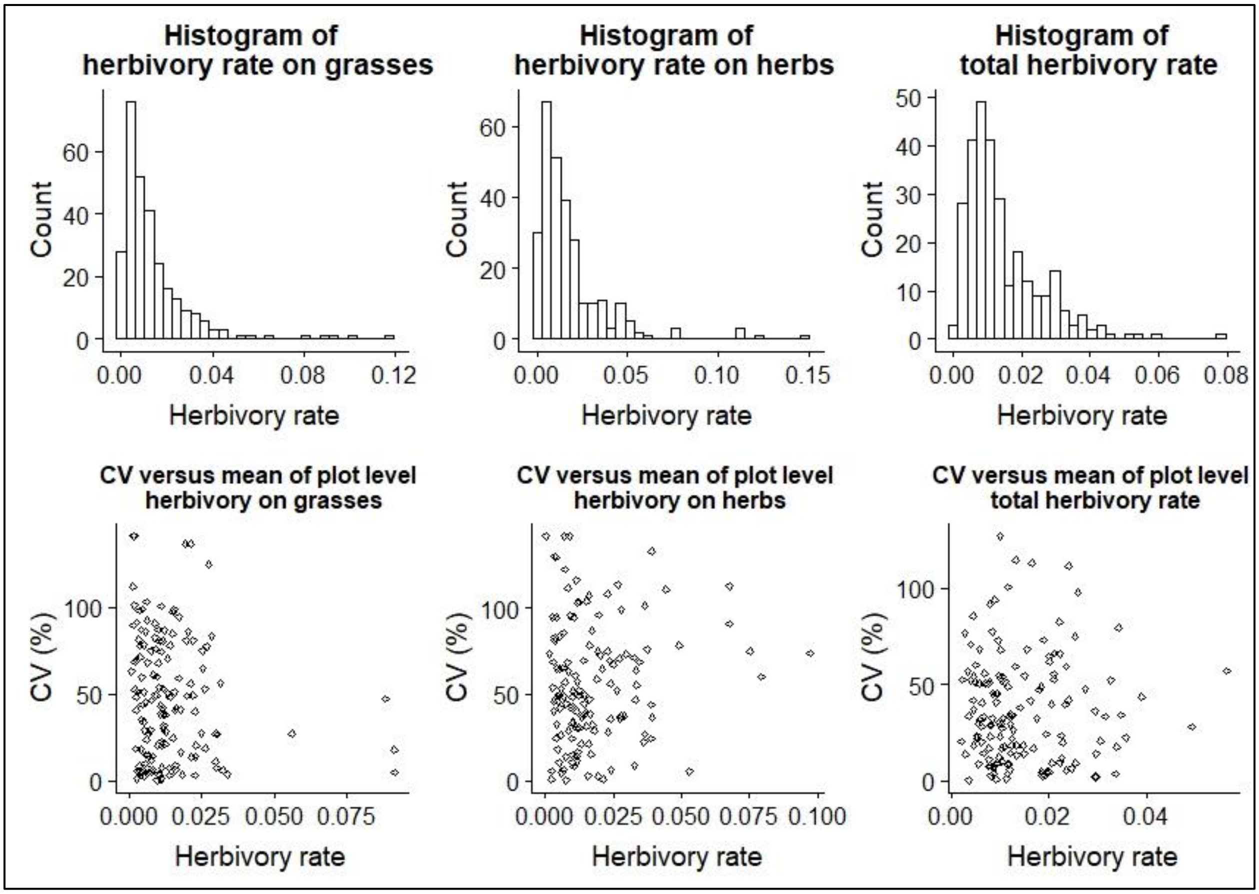

Figure S29 top: histograms showing subplot level herbivory rates in grasslands; bottom: scatterplots showing plot level mean rates and CV

References

Blüthgen N, Dormann CF, Prati D, Klaus VH, Kleinebecker T, Hölzel N, Alt F, Boch S, Gockel S, Hemp A, Müller J, Nieschulze J, Renner SC, Schöning I, Schumacher U, Socher SA, Wells K, Birkhofer K, Buscot F, Oelmann Y, Rothenwöhrer C, Scherber C, Tscharntke T, Weiner CN, Fischer M, Kalko EKV, Linsenmair KE, Schulze E-D, Weisser WW. 2012. A quantitative index of land-use intensity in grasslands: Integrating mowing, grazing and fertilization. Basic and Applied Ecology 13: 207-220.

Boch S, Prati D, Müller J, Socher S, Baumbach H, Buscot F, Gockel S, Hemp A, Hessenmöller D, Kalko EK. 2013. High plant species richness indicates management-related disturbances rather than the conservation status of forests. Basic and Applied Ecology 14: 496-505.

Gossner MM, Weisser WW, Meyer ST. 2014. Invertebrate herbivory decreases along a gradient of increasing land-use intensity in German grasslands. Basic and Applied Ecology 15: 347-352.

Kahl T, Bauhus J. 2014. An index of forest management intensity based on assessment of harvested tree volume, tree species composition and dead wood origin. Nature Conservation 7: 15-27.

Kassambara A, Mundt F. 2017. Factoextra: Extract and Visualize the Results of Multivariate Data Analyses. pR package version 1.0.5.

Loranger H, Weisser WW, Ebeling A, Eggers T, De Luca E, Loranger J, Roscher C, Meyer ST. 2014. Invertebrate herbivory increases along an experimental gradient of grassland plant diversity. Oecologia 174: 183-193.

R Core Team R. 2017. R: A language and environment for statistical computing. Vienna, Austria: R Foundation for Statistical Computing.

Vu V. 2011. Ggbiplot v.0.55: A ggplot2 based biplot.
